# Comparison of drug approvals of the FDA and EMA between 2013 and 2023

**DOI:** 10.1007/s00210-025-04412-4

**Published:** 2025-07-03

**Authors:** Franziska Lau, Roland Seifert

**Affiliations:** Institute of Pharmacology, Hannover Medical School, Carl-Neuberg-Str. 1, Hannover, D-30655 Germany

**Keywords:** Drug regulation, Novel drug approvals, European medicines agency (EMA), US food and drug administration (FDA), New active substance (NAS), New molecular entity (NME)

## Abstract

To what extent do drug approvals differ between Europe (EMA) and the USA (FDA) differ, and how do these differences arise? The study examines differences in approval timelines, therapeutic areas, mechanisms of action, and the marketing authorisation holders for the period between 2013 and 2023. All medicines containing a new active substance (NAS) from the European Medicines Agency’s (EMA) Annual Reports, as well as all new molecular entities (NMEs) from the US Food and Drug Administration’s (FDA) New Drug Therapy Approvals Reports and the annual Biological Approvals were assessed. The FDA authorised significantly more novel drugs (583) than the EMA (424) during the analysed period. The FDA granted far more exclusive drug approvals (185) than the EMA (42). Both agencies prioritise the therapeutic areas oncology, infectious diseases, haematology, and neurology. Enzyme inhibitors and antibodies are the most common drug classes. Pfizer, Novartis, and AstraZeneca are the Top-3 marketing authorisation holders (MAHs) for the FDA and EMA. The overall US share of EMA approvals exceeds the EU share of FDA approvals. The FDA also tends to use faster and more flexible approval pathways and shows greater tolerance for uncertainty in benefit-risk assessments. It also relies more frequently on surrogate endpoints and limited clinical data, particularly in accelerated approval pathways. In contrast, the EMA shows a stronger focus on long-term safety and public health priorities. Minor timing differences were observed, with the FDA generally authorising drugs earlier. These differences reflect not only company strategies and geographic patterns but also divergent regulatory processes due to different financial frameworks. The FDA also works with a broader range of MAHs overall, suggesting a more diverse and globally active company base. It also approves a wider variety of drugs, including more drugs with unknown mechanism of action. Various international initiatives aim to harmonise regulatory approaches and promote collaboration between agencies. Despite these efforts, differences remain, especially in response to urgent public health challenges. The FDA takes a more exploratory approach to drug approvals, whereas the EMA is more public health oriented. Differences in financial framework, decision-making processes, therapeutic priorities, geographical factors, and strategic marketing decisions of drug companies all contribute to the differences observed in this study. Efforts are in progress to globally harmonise drug approval processes.

**Supplementary Information:**

The online version contains supplementary material available at 10.1007/s00210-025-04412-4.

## Introduction

The approval of pharmaceuticals plays an important role in public health by ensuring that new therapies are safe, effective, and of high quality before they become available to the population. Differences in the approval processes have a significant impact on patient access to new therapies in different regions and may complicate global harmonisation efforts. Especially the COVID-19 pandemic showed that new therapeutics are not always approved simultaneously across all regions. For example, the COVID-19 vaccine Spikevax was authorised by the EMA in December 2021 and by the FDA in January 2022 (European Medicines Agency 2021a; US Food and Drug Administration 2022a). Some drugs may not get authorised in certain regions at all.

In Europe, the EMA is responsible for evaluating, approving, and monitoring the safety, efficacy, and quality of medicines to ensure they meet the highest standards for public health (https://www.ema.europa.eu/en/about-us/what-we-do, last accessed March 6, 2025). In the USA, the FDA is responsible for regulating, approving, and monitoring pharmaceuticals, medical devices, and other health-related products to ensure they are safe, effective, and of high quality for public use (https://www.fda.gov/about-fda/what-we-do#mission, last accessed March 6, 2025). These two influential regulatory authorities play a fundamental role in deciding which medicines are available in their respective markets and in monitoring drug safety beyond initial approval.

Previous studies have examined the approval processes of the EMA and FDA from different perspectives. Table 1 provides an overview of relevant publications on this topic. While these studies provide valuable insights into specific regulatory aspects, there is currently no systematic comparison of approval timelines, mechanisms of action, therapeutic areas, and MAHs of NMEs covering an extended time frame.

Therefore, this study is aimed at systematically comparing drug approvals by the EMA and FDA between 2013 and 2023 based on approval timelines, regulatory frameworks, therapeutic focus, and marketing authorisation strategies.

**Table 1.** Selection of previously published literature on the comparison of drug approvals between the EMA and the FDA

Author(s)	Year	Title	Journal/source	Methodology	Relevance to comparison
**Zeitoun et al. **^a^	2017	Postmarketing studies for novel drugs approved by both the FDA and EMA between 2005 and 2010: a cross-sectional study	BMJ Open	Cross-sectional analysis of post-marketing studies	Highlights the variability and coordination challenges in post-marketing research between the FDA and EMA
**Kashoki et al.**^b^	2019	A Comparison of EMA and FDA Decisions for New Drug Marketing Applications 2014–2016: Concordance, Discordance, and Why	Clinical Pharmacology and Therapeutics	Comparative analysis of 107 new drug applications	Shows high concordance (91–98%) in decisions, differences mostly due to efficacy interpretation
**Hwang et al.**^c^	2020	Association between FDA and EMA expedited approval programs and therapeutic value of new medicines: retrospective cohort study	The BMJ	Retrospective cohort study	Explores how expedited programmes influence the approval process
**Downing et al.**^d^	2021	Assessment of FDA and EMA-approved systemic oncology therapies and Clinically Meaningful Improvements in Quality of Life	JAMA Network Open	Systematic review of oncology drug approvals	Provides insights on differences in approval criteria for cancer drugs and clinically meaningful improvements in quality of life
**Vokinger et al.**^e^	2022	Therapeutic Value of Drugs Granted Accelerated Approval or Conditional Marketing Authorization in the US and Europe From 2007 to 2021	JAMA Health Forum	Cohort study analysing drugs granted Accelerated Approval in the US or conditional marketing authorization in the EU	Highlights regulatory differences between FDA and EMA regarding Accelerated Approvals, emphasising post-approval study completion needs
**Lythgoe et al.**^f^	2022	Cancer Therapy Approval Timings, Review Speed, and Publication of Pivotal Registration Trials in the US and Europe, 2010–2019	JAMA Network Open	Cross-sectional study of oncology therapy approvals	Provides comparative insights on how FDA and EMA differ in approval speeds for cancer drugs
**Papapetropoulos et al.**^g^	2023	Novel drugs approved by the EMA, the FDA, and the MHRA in 2023	British Journal of Pharmacology	Review of 70 novel drugs approved in 2023	Provides a recent snapshot of FDA and EMA approvals, emphasising cutting-edge technologies

EMA and the FDA

^a^Zeitoun et al. (2017)

^b^Kashoki et al. (2019)

^c^Hwang et al. (2020)

^d^Downing et al. (2021)

^e^Vokinger et al. (2022)

^f^Vokinger et al. (2022)

^g^Papapetropoulos et al. (2023)

## Materials and methods

All novel drugs approved by the EMA and FDA between 2013 and 2023 were included in the study. Only EMA approvals authorised through the EU centralised procedure were considered. National or decentralised procedures were excluded.

In this study, the term ‘novel drugs’ includes therapeutics that are classified as NME by the FDA or defined as NAS by the EMA. Although the definitions are broadly aligned, the EMA focuses more on therapeutic and structural novelty, whereas the FDA distinguishes NMEs and ‘new chemical entities’ (NCE) for legal and regulatory purposes. Overall, these terms refer to drugs that contain active compounds that have not been previously authorised in the USA or EU. This applies both to the active ingredient on its own and as part of combination products (https://www.fda.gov/drugs/development-approval-process-drugs/novel-drug-approvals-fda, last accessed March 10, 2025; European Medicines Agency 2015a).

To analyse and compare these novel drug approvals in the USA and EU between 2013 and 2023, three main sources were used:

A) The ‘Annual Report’, published annually by the EMA on its official website (https://www.ema.europa.eu/en/about-us/annual-reports-work-programmes, last accessed September 30, 2024)
B) The ‘New Drug Therapy Approvals Report’ published annually by the FDA on its official website (https://www.fda.gov/drugs/development-approval-process-drugs/novel-drug-approvals-fda, last accessed October 28, 2024)
C) The ‘Biological License Application Approvals’ published annually by the FDA on its official website (https://www.fda.gov/vaccines-blood-biologics/development-approval-process-cber/biological-approvals-year, last accessed October 28, 2024).This source was included because, unlike the EMA, the FDA publishes novel biological drugs such as vaccines, gene therapy drugs, and related products separately from other approvals. Blood products, plasma derivatives, immunoassays, and similar products were excluded from this analysis.

It is important to note that the EMA can only recommend authorisation by issuing a positive opinion. The final decision for a legally binding authorisation lies with the European Commission (https://www.ema.europa.eu/en/about-us/what-we-do/authorisation-medicines, last accessed March 6, 2025).

Excel tables were created including the following categories: active substance, commercial name (trade name), mechanism of action, therapeutic area, MAH, and the year of approval. Based on these categories, the drug approvals of both agencies could be analysed and compared.

During the analysed period, the EMA approved a total of 424 new drugs, while the FDA approved 583 drugs. Among these, 42 drugs were approved only by the EMA and 185 only by the FDA (agency-exclusive approvals). Three hundred forty-seven drugs received approval from both agencies within the analysed time frame. The remaining approvals were either granted approval by the other agency before the study period or do not appear in the reports, for example, due to alternative approval pathways or reintroduction of previously approved molecular entities.

**Fig. 1:**
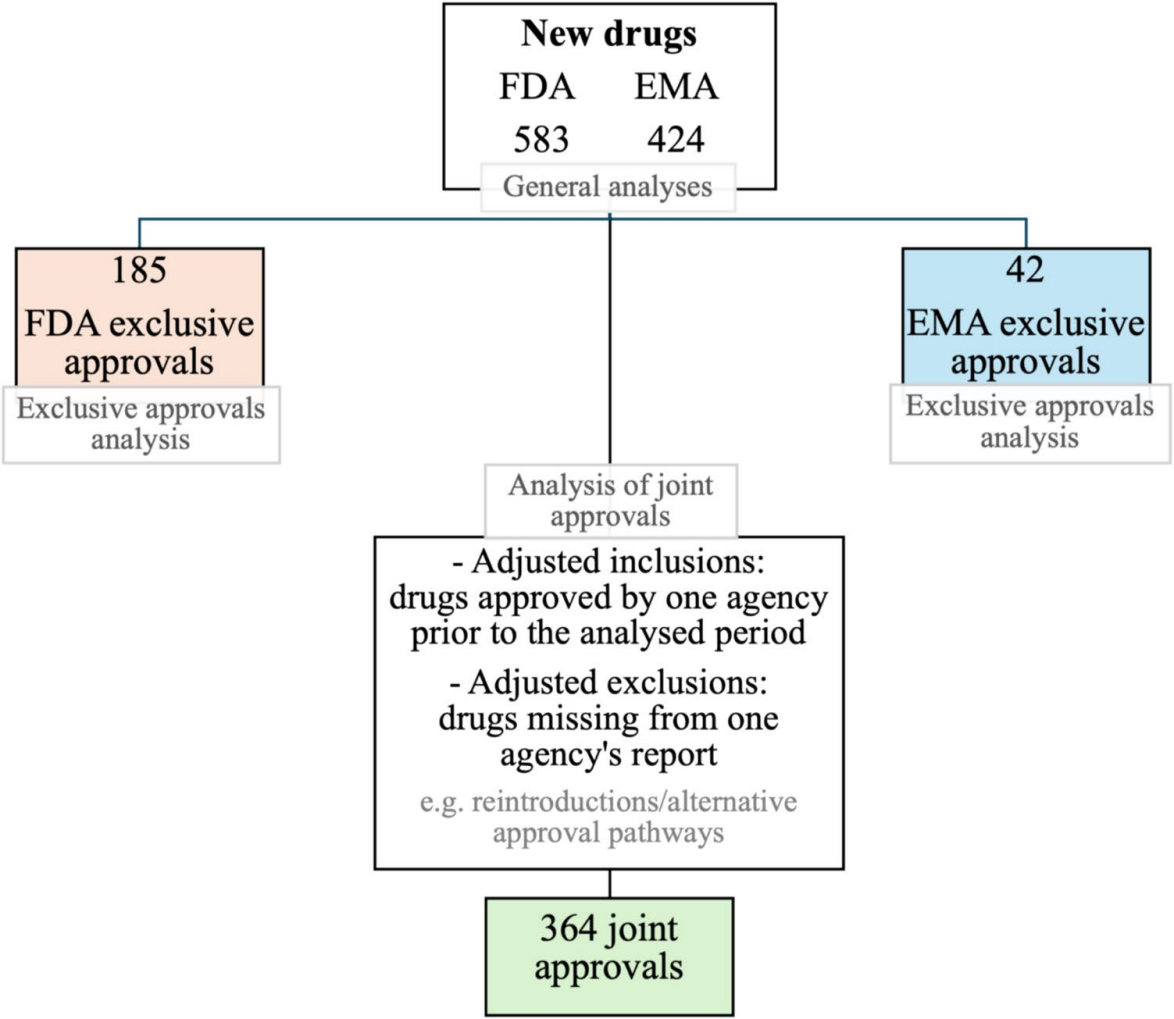
Overview of FDA and EMA drug approvals from 2013 to 2023 and their classification into exclusive and joint approvals based on adjustments for prior authorisations and reporting discrepancies to ensure accurate grouping

For the analysis of approval timing for joint approvals, not only drugs approved within the analysed period were considered but also cases in which one agency approved the drug prior to the defined timeframe. Therefore, a misclassification as agency-exclusive could be avoided. Drugs that were approved by both agencies but missing from one agency’s report were excluded from this joint approval analysis, as modified approval pathways or reintroductions of active moieties could bias the comparison of approval timing. Figure 1 provides a visual overview of this classification process.

## Results

### Number of novel drug approvals from 2013 to 2023

**Fig. 2:**
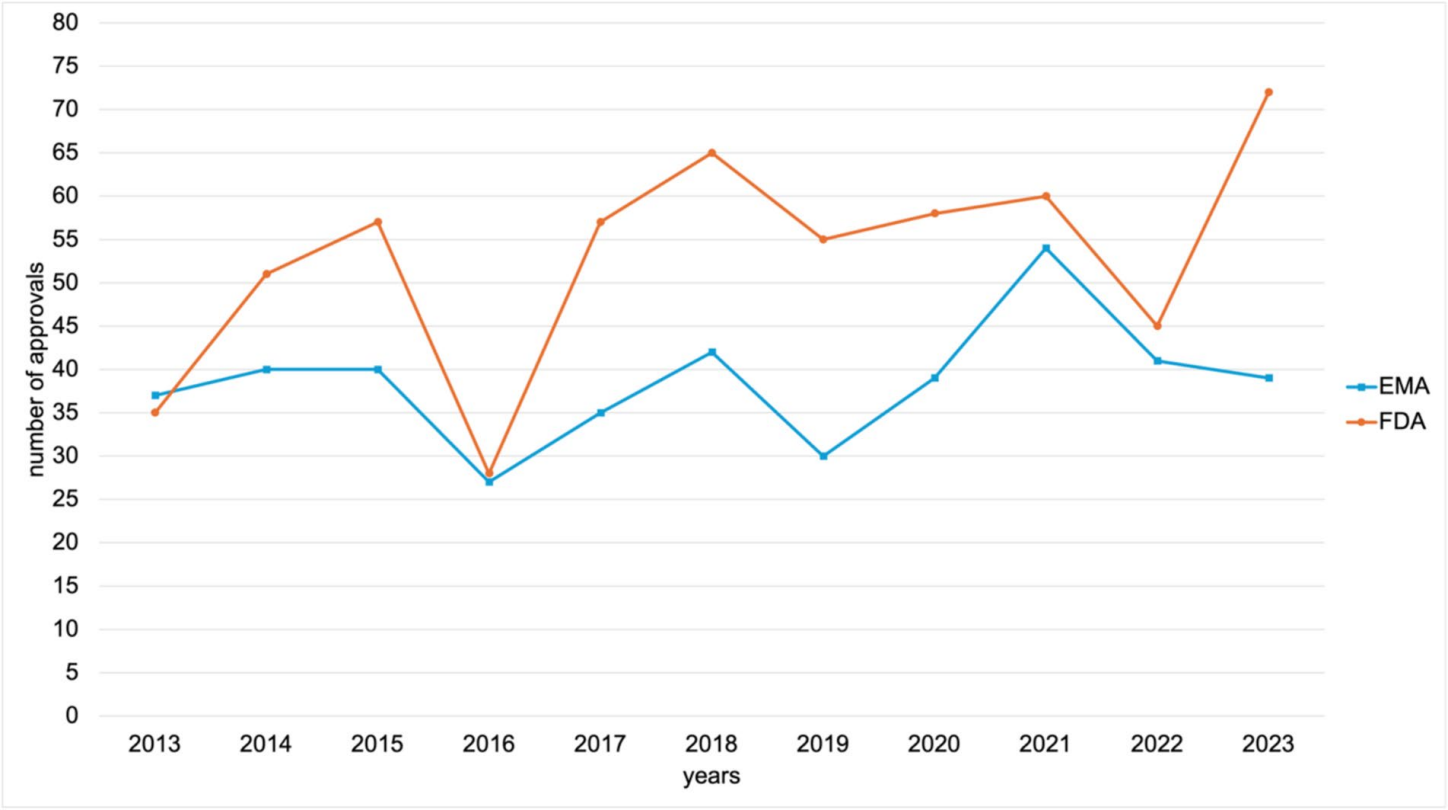
Comparison of the number of EMA and FDA novel drug approvals from 2013 to 2023, shown in a line chart

Figure 2 provides an overview of the annual number of novel drug approvals by the EMA and FDA over the analysed period.

In total, the FDA approved 583 new molecular entities. The highest number was recorded in 2023, with 72 approvals, followed by 65 in 2018 and 60 in 2021. The lowest number of approvals was recorded in 2016, with only 28. In 2013, the FDA authorised 35 drugs, and in 2022, 45 were approved.

The EMA approved a total of 424 novel drugs. The highest number of EMA approvals was recorded in 2021, with 54 drugs. This was followed by 42 approvals in 2018 and 41 in 2022.

The lowest number of approvals was recorded in 2016 with 27, while 30 drugs were authorised in 2019 and 35 in 2017.

**Fig. 3:**
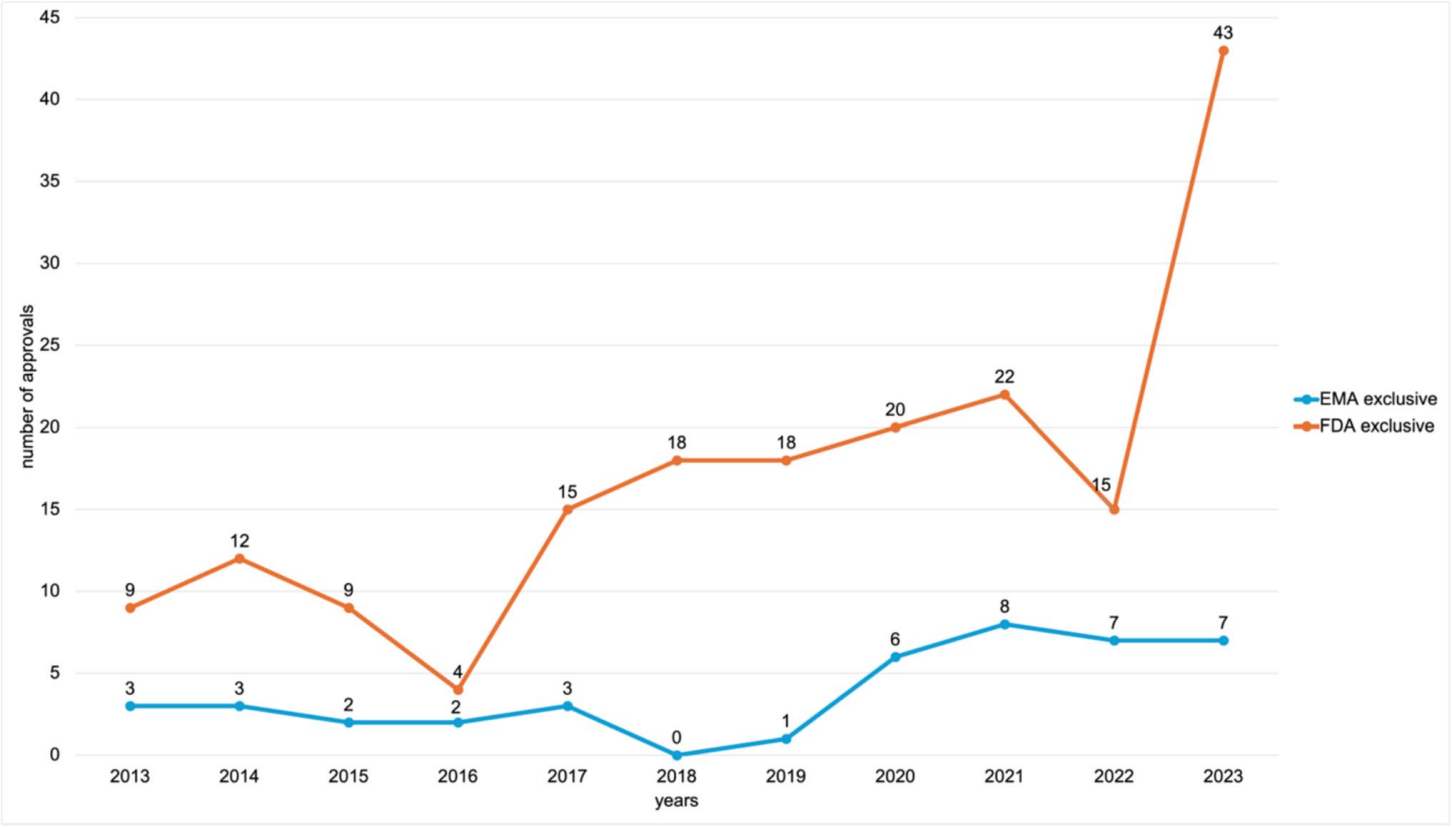
Comparison of the number of EMA and FDA exclusive novel drug approvals from 2013 to 2023, shown in a line chart

The annual numbers of these agency-exclusive approvals are shown in Fig. 3. Overall, the FDA authorised a total of 185 exclusive drugs. The highest number occurred in 2023, with 43 approvals, followed by 22 in 2021, 20 in 2020, and 18 in both 2018 and 2019. The lowest number was recorded in 2016, with only 4 exclusive approvals. In 2013 and 2015, the FDA approved 9 exclusive drugs each, and in 2014, 12 approvals were granted. The EMA authorised a total of 42 exclusive drugs during this period. Its highest number occurred in 2021, with 8 approvals, followed by 2023 and 2022 with 7 approvals each, and 2020 with 6. In 2013, 2014, and 2017, 3 drugs were authorised per year. In 2015 and 2016, only 2 drugs were approved each, while in 2019, just 1 was authorised. No exclusive EMA approvals were recorded in 2018.

### Therapeutic areas of novel drug approvals from 2013 to 2023

Table 2 presents a total overview of therapeutic areas of novel drug approvals by the EMA and FDA. It includes 424 EMA approvals and 583 FDA approvals. Supplementary Fig. 1 shows the same data as relative percentages, allowing for a direct comparison despite the differing total numbers of approvals. For both agencies, most drugs were authorised in the therapeutic area malignant diseases, with 124 approvals by the FDA and 114 by the EMA. The following categories are similar for both agencies, though in a different order: The FDA approved 71 drugs in haematology/haemostaseology, 66 in neurology, and 63 in infectious diseases. In contrast, the EMA approved 49 drugs in infectious diseases, 43 in haematology/haemostaseology, and 38 in neurology. The least represented therapeutic areas for the FDA were reproductive medicine and plastic surgery/aesthetic medicine with 3 approvals each, followed by toxicology, musculoskeletal system, and COVID-19, with 2 approvals each. Pain and ‘other’ were each represented by 1 approval. In contrast, the EMA approved 3 drugs each in the categories reproductive medicine and psychiatry, 1 drug each in musculoskeletal system and ‘other’, while no approvals were recorded in plastic surgery/aesthetic medicine, toxicology, or pain. Overall, the trends are quite similar, given the overall higher number of FDA approvals. However, the therapeutic area COVID-19, with 13 EMA and 2 FDA approvals, differs from the overall trend, as it is the only category with more approvals by the EMA than by the FDA.

**Table 2:** Therapeutic areas of EMA and FDA novel drug approvals from 2013 to 2023

Therapeutic areas	EMA approvals	FDA approvals
Malignant disease	114	124
Infections	49	63
Haematology/haemostaseology	43	71
Neurology	38	66
Metabolism	25	27
Endocrinology	24	30
Immunology/rheumatology/transplantation	20	31
Vaccines	17	29
Dermatology	16	22
Pneumology/allergology	13	19
Cardiovascular	13	16
COVID-19	13	2
Gastro./hepatology	9	17
Ophthalmology	8	14
Uro-nephrology	8	11
Diagnostic agents	6	18
Psychiatry	3	11
Reproductive medicine	3	3
Musculoskeletal system	1	2
Other	1	1
Plastic surgery/aesthetic medicine	0	3
Toxicology	0	2
Pain	0	1

**Fig. 4:**
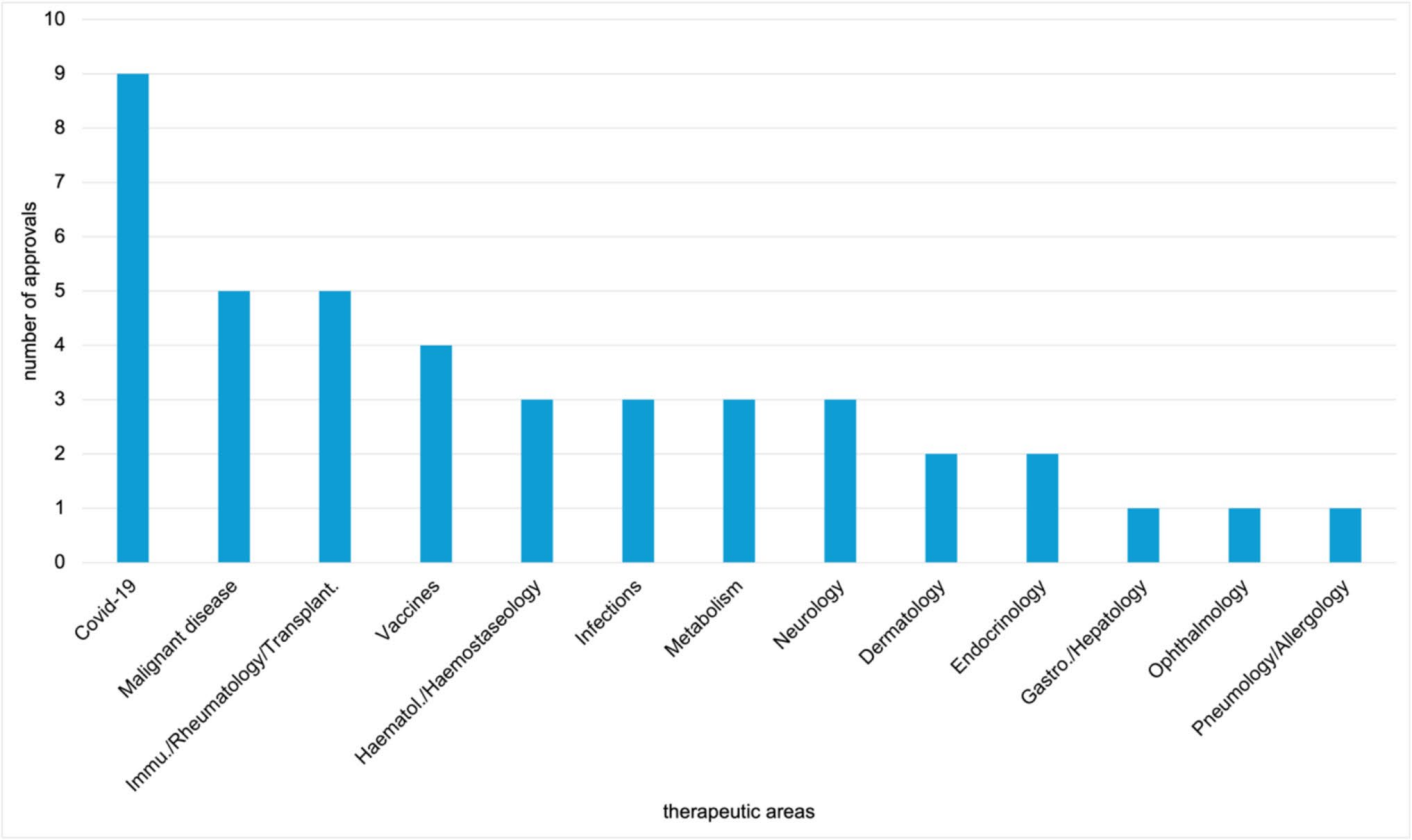
Therapeutic areas of EMA exclusive novel drug approvals from 2013 to 2023, shown in a bar chart

Figure 4 shows the distribution of therapeutic areas for EMA-exclusive approvals. The overview includes 42 drugs, assigned to 13 therapeutic areas. Nine drugs were authorised for COVID-19, 5 each in malignant disease and immunology/rheumatology/transplantation, and 4 in vaccines. Haematology/haemostaseology, infections, metabolism, and neurology are each represented by 3 approvals, dermatology and endocrinology by 2 each, and gastroenterology/hepatology, ophthalmology, and pneumology/allergology by one each.

**Fig. 5:**
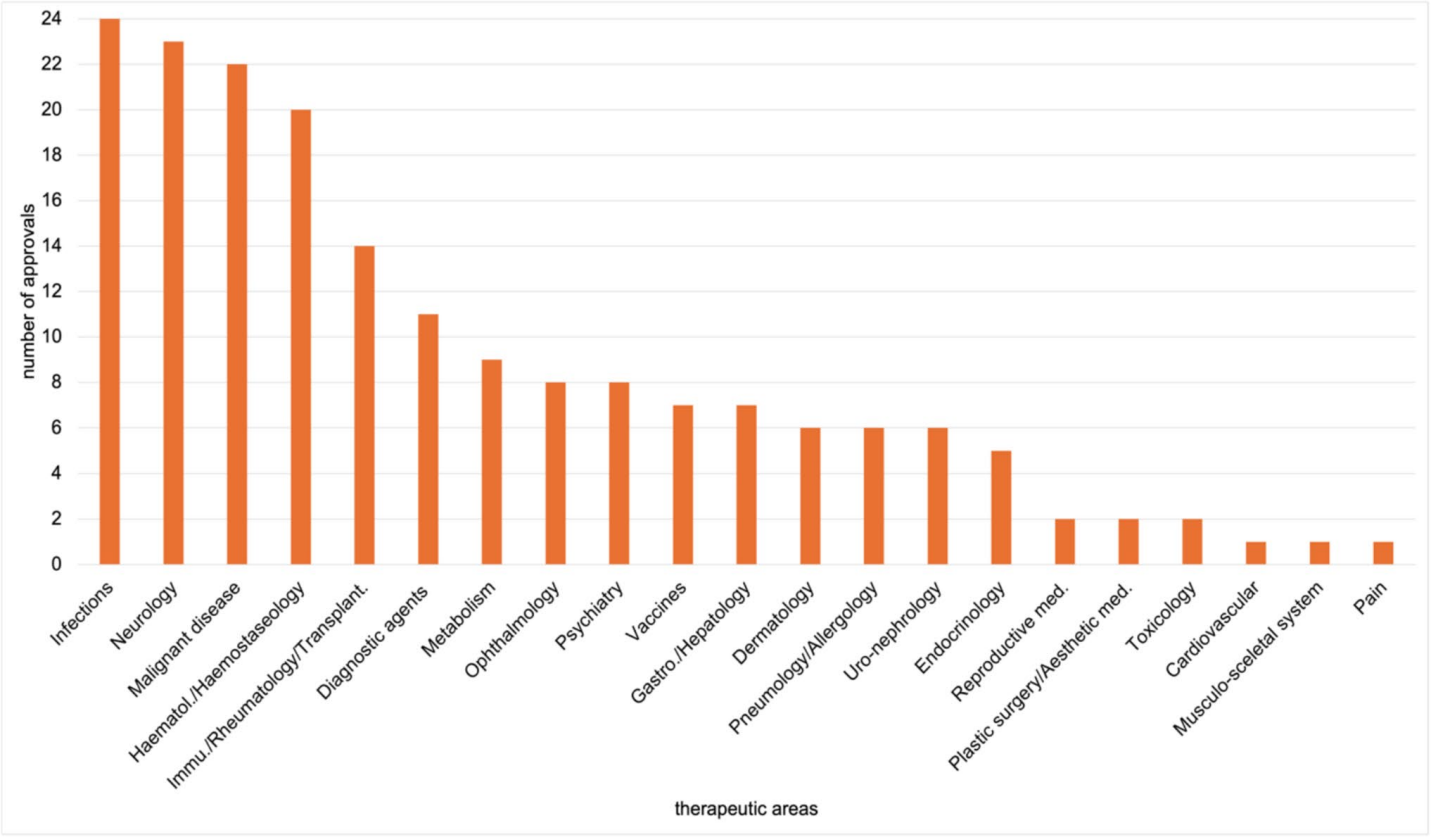
Therapeutic areas of FDA exclusive novel drug approvals from 2013 to 2023, shown in a bar chart

As shown in Fig. 5, the 185 exclusive FDA approvals span 21 therapeutic areas. While COVID-19 is the only category represented exclusively by the EMA, 9 therapeutic areas are exclusive to the FDA: diagnostic agents with 11, psychiatry with 8, uro-nephrology with 6, and cardiovascular and reproductive medicine each with 1 approval. Plastic surgery/aesthetic medicine (2), toxicology (2), musculoskeletal system (1), and pain (1) are exclusive to the FDA. Most FDA-exclusive drugs were authorised in the therapeutic area infections with 24, followed by neurology with 23, malignant disease with 22, and haematology/haemostaseology with 20 approvals. Supplementary Figs. 2–5 present these data as relative percentages.

### Mechanisms of action of novel drug approvals from 2013 to 2023

Table 3 presents an overview of mechanisms of action among novel drug approvals. In total, 424 EMA approvals were classified into 30 different mechanisms, while 583 FDA approvals span 29 mechanisms. To improve clarity, the table shows the 15 most frequently authorised mechanisms and therefore represents a subset of 393 EMA and 552 FDA approvals. For both agencies, most authorised drugs are enzyme inhibitors, with 139 approvals by the FDA and 123 by the EMA. The following most common mechanisms are similar for both agencies: antibodies, with 72 EMA and 75 FDA approvals, and replacement therapy/substitution/transplantation, with 32 EMA and 68 FDA approvals. Beyond these categories, the orders differ. The FDA approved 49 drugs with unclear mechanisms of action, 45 receptor antagonists, and 38 receptor agonists. In contrast, the EMA authorised 32 receptor antagonists, 25 vaccines, and 23 receptor agonists. Among the least frequent mechanisms shown in the figure, the FDA approved 5 protein modulators, 7 immunomodulators, and 10 antibody–drug conjugates. For the EMA, the lowest number was recorded for receptor modulators, with 5 approvals, followed by protein modulators and RNA/DNA modifiers, with 7 each. Immunomodulators and channel/transport inhibitors were slightly more common, with 8 approvals each. These numbers are shown in relative percentages in Supplementary Fig. 6.

**Table 3:** Top 15 mechanisms of action of EMA and FDA novel drug approvals from 2013 to 2023

Mechanisms of action	EMA approvals	FDA approvals
Enzyme inhibitors	123	139
Antibody	72	75
Replacement therapy/substitution/transplantation	32	68
Receptor antagonists	32	45
Vaccines	25	29
Receptor agonists	23	38
Gene-/cell therapy	20	22
Unclear	12	49
Diagnostic agents/contrast agents	10	22
Antibody–drug conjugates	9	10
Channel-/transport inhibitors	8	14
Immunomodulators	8	7
RNA/DNA modification	7	16
Protein modulators	7	5
Receptor modulators	5	13

**Fig. 6:**
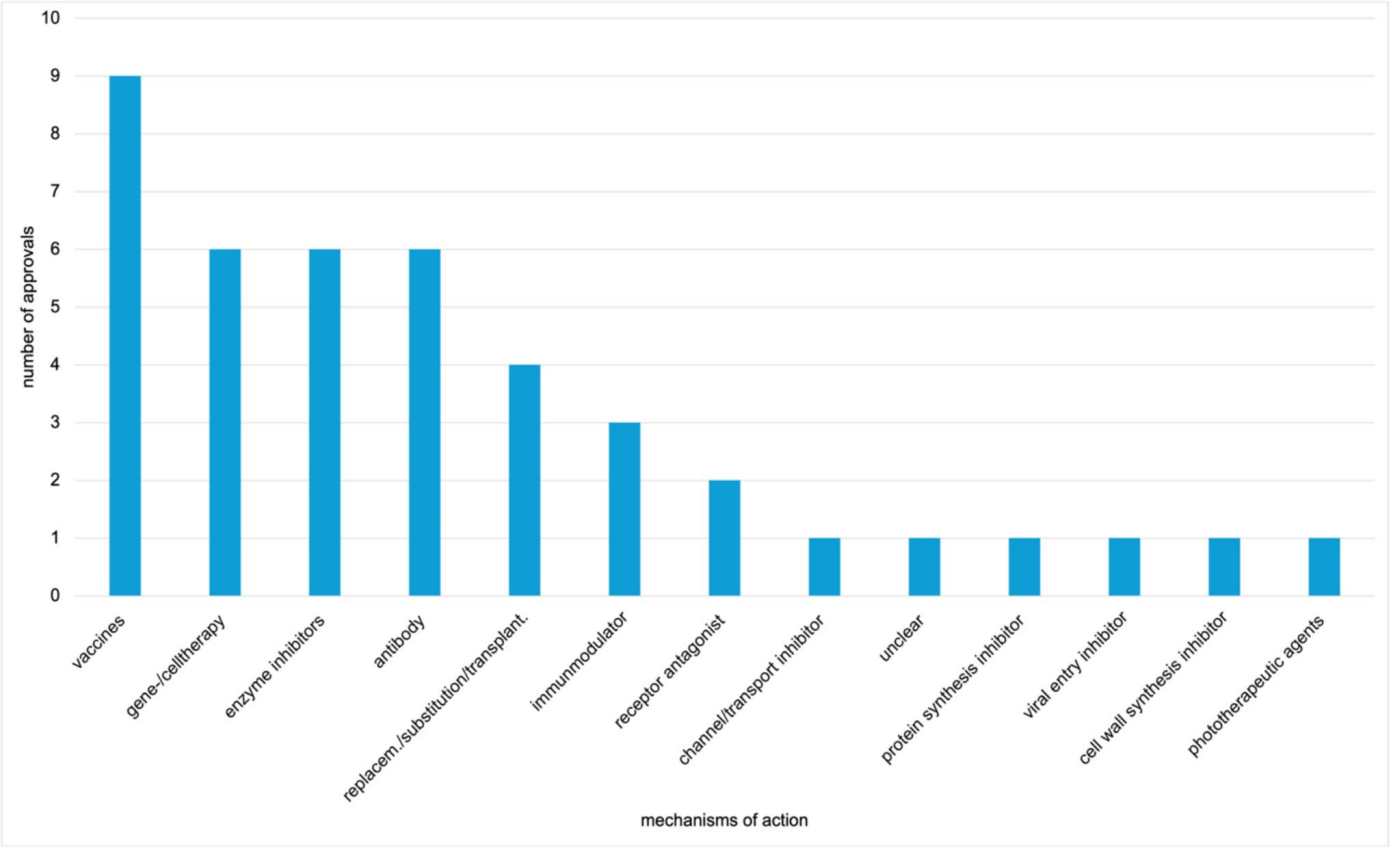
Mechanisms of action of EMA exclusive novel drug approvals from 2013 to 2023, shown in a bar chart

Figure 6 shows the 42 EMA-exclusive approvals, classified under 13 mechanisms of action. Vaccines accounted for the highest number, with 9 approvals, followed by gene-/cell therapy, enzyme inhibitors, and antibodies, with 6 each. Four drugs were authorised under replacement/substitution/transplantation therapies, 3 as immunomodulators, and 2 as receptor antagonists. Six categories were represented by 1 approval each: channel/transport inhibitors, drugs with unclear mechanisms, protein synthesis inhibitors, viral entry inhibitors, cell wall synthesis inhibitors, and phototherapeutic agents.

**Fig. 7:**
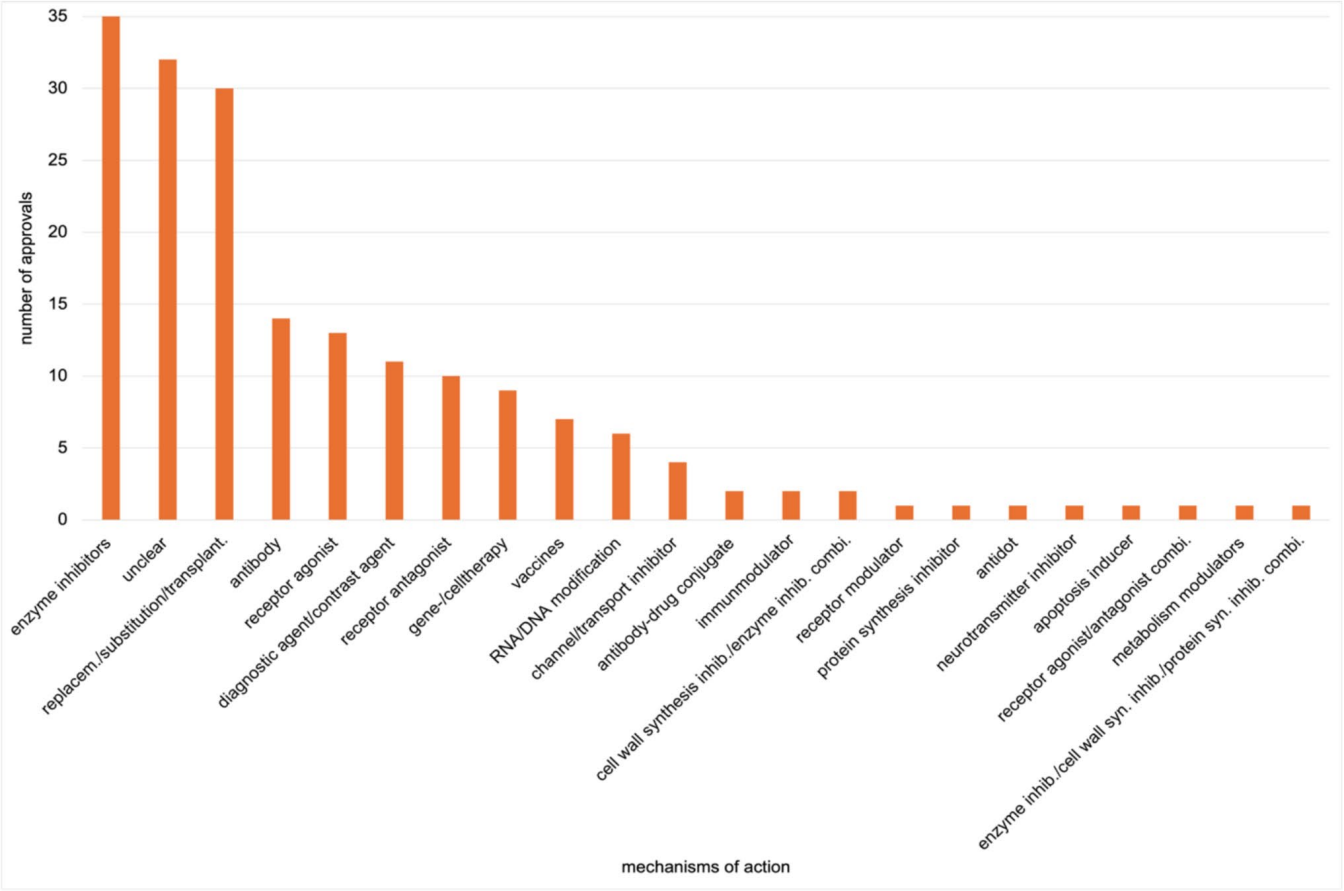
Mechanisms of action of FDA exclusive novel drug approvals from 2013 to 2023, shown in a bar chart

In contrast, the 185 FDA-exclusive approvals span 22 mechanisms of action, as shown in Fig. 7. While viral entry inhibitors, cell wall synthesis inhibitors, and phototherapeutic agents appear exclusively in EMA approvals, 11 mechanisms are exclusive to the FDA. These include receptor agonists, diagnostic/contrast agents, RNA/DNA modifiers, antibody–drug conjugates, receptor modulators, antidots, neurotransmitter inhibitors, apoptosis inducers, metabolism modulators, and two combination products.

With 35 approvals, enzyme inhibitors were the most frequently authorised category among FDA-exclusive approvals. This aligns with the distribution of overall FDA approvals. Thirty-two drugs had an unclear mechanism of action, and 30 fell under replacement/substitution/transplantation therapies.

Eight mechanisms were each represented by just one drug: receptor modulators, protein synthesis inhibitors, antidots, neurotransmitter inhibitors, apoptosis inducers, metabolism modulators, receptor agonist/antagonist combinations, and a triple combination of enzyme inhibitor/cell wall inhibitor/protein synthesis inhibitor. Mechanisms of action as relative percentages are shown in Supplementary Figs. 10 and 11. Supplementary Figs. 12 and 13 show mechanisms of overall and exclusive approvals for the EMA and FDA.

### Analysis of marketing authorisation holders from 2013 to 2023

In total, 124 MAHs hold the authorisations for 424 EMA approvals, while 583 FDA approvals span 214 MAHs. Table 4 presents the 15 most frequently represented MAHs among overall drug approvals by each authority. Overall, these most common MAHs are similar for the EMA and FDA, though in different orders. For both authorities, the top three MAHs are the same: Pfizer, with 23 EMA and 28 FDA approvals; Novartis, with 22 EMA and 27 FDA approvals; and AstraZeneca, with 21 approvals from each authority. For the EMA, Merck Sharp and Dohme (MSD) and Roche follow, each with 19 approvals. Amgen ranks lowest, with only 8 approvals, followed by Bayer with 9 and AbbVie with 10. In contrast, for the FDA, Sanofi follows the top three MAHs, with 20 approvals, followed by Bristol Myers Squibb with 19. The fewest authorisations in this figure are held by Bayer, with 9, followed by Amgen, with 11 and Gilead Sciences, with 12 approvals. Supplementary Figs. 11 and 12 show the distribution of marketing authorisation holders as relative percentages for both the EMA and FDA.

**Table 4:** Top 15 marketing authorisation holders of EMA and FDA novel drug approvals from 2013 to 2023

Marketing authorisation holders	EMA approvals	FDA approvals
Pfizer	23	28
AstraZeneca	21	21
Merck Sharp Dome (MSD)	19	18
Roche	19	17
Novartis	18	27
Johnson & Johnson	16	16
Gilead Sciences	16	12
Bristol Meyers Squibb	13	19
GSK (GlaxoSmithKline)	13	16
Eli Lilly	13	14
Sanofi	12	20
Takeda Pharmaceutical	12	18
AbbVie	10	15
Bayer	9	9
Amgen	8	11

Most of the top-15 MAHs are headquartered in the USA. These include Pfizer, MSD, Gilead Sciences, Bristol Myers Squibb, Johnson & Johnson, AbbVie, Eli Lilly, and Amgen. Fewer are based in the EU, including the UK, as this analysis covers data from years prior to Brexit in 2020: Sanofi (France), Bayer (Germany), AstraZeneca (UK), and GSK (UK). Furthermore, Takeda Pharmaceutical (Japan), Novartis (Switzerland), and Roche (Switzerland) are headquartered outside both the EU and the USA.

**Fig. 8:**
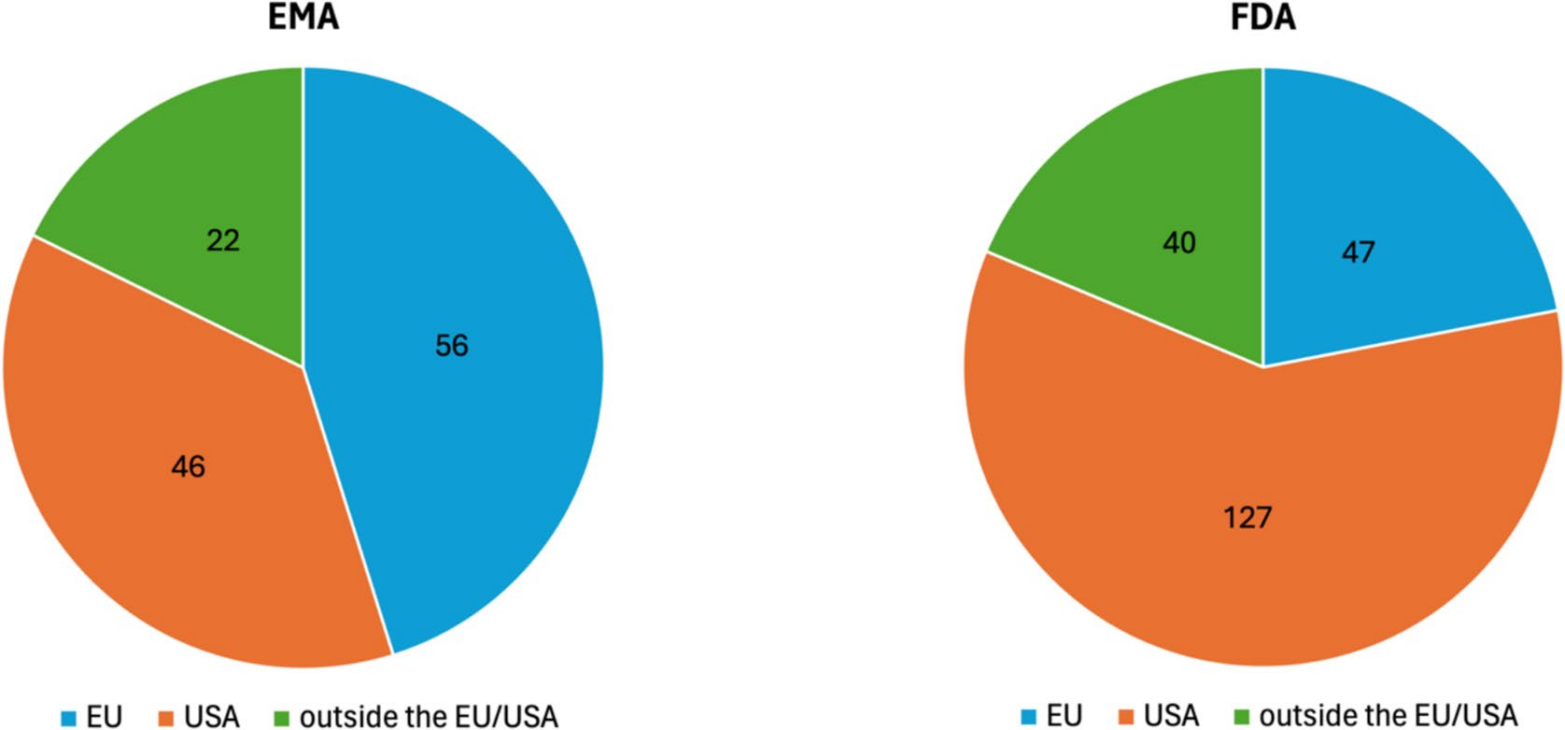
Distribution of company headquarters of marketing authorisation holders from 2013 to 2023, based on the total number of authorisation holders, shown in two pie charts

To further examine this observation, Fig. 8 shows the distribution of MAH headquarters over the analysed period. Among EMA MAHs, 56 are based in the EU, 46 in the USA, and 22 outside the EU and USA. In contrast, 127 FDA MAHs are based in the USA, 47 in the EU, and 40 outside the EU and USA. These findings show that both agencies grant most approvals to companies based in their respective region and fewest to those based elsewhere. However, the proportion of US-based companies among FDA approvals is higher than the proportion of EU-based companies among EMA approvals. Additionally, the share of US-based companies among EMA approvals exceeds that of EU-based companies among FDA approvals. The data indicate a stronger regional focus in the FDA’s approvals compared to those of the EMA. Supplementary Fig. 13 provides the same data as percentages.

**Fig. 9:**
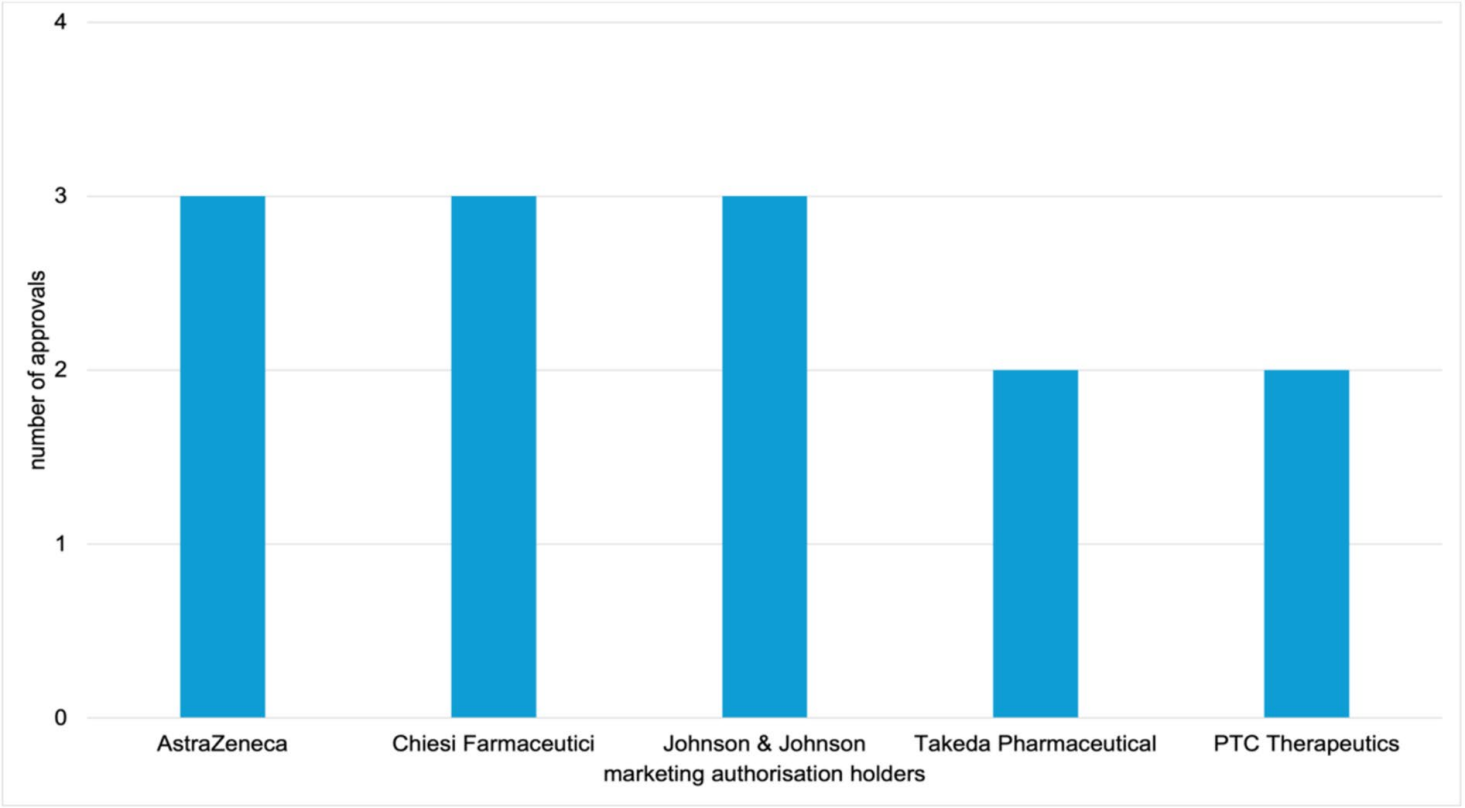
Top 5 marketing authorisation holders of EMA exclusive novel drug approvals from 2013 to 2023, shown in a bar chart

**Fig. 10:**
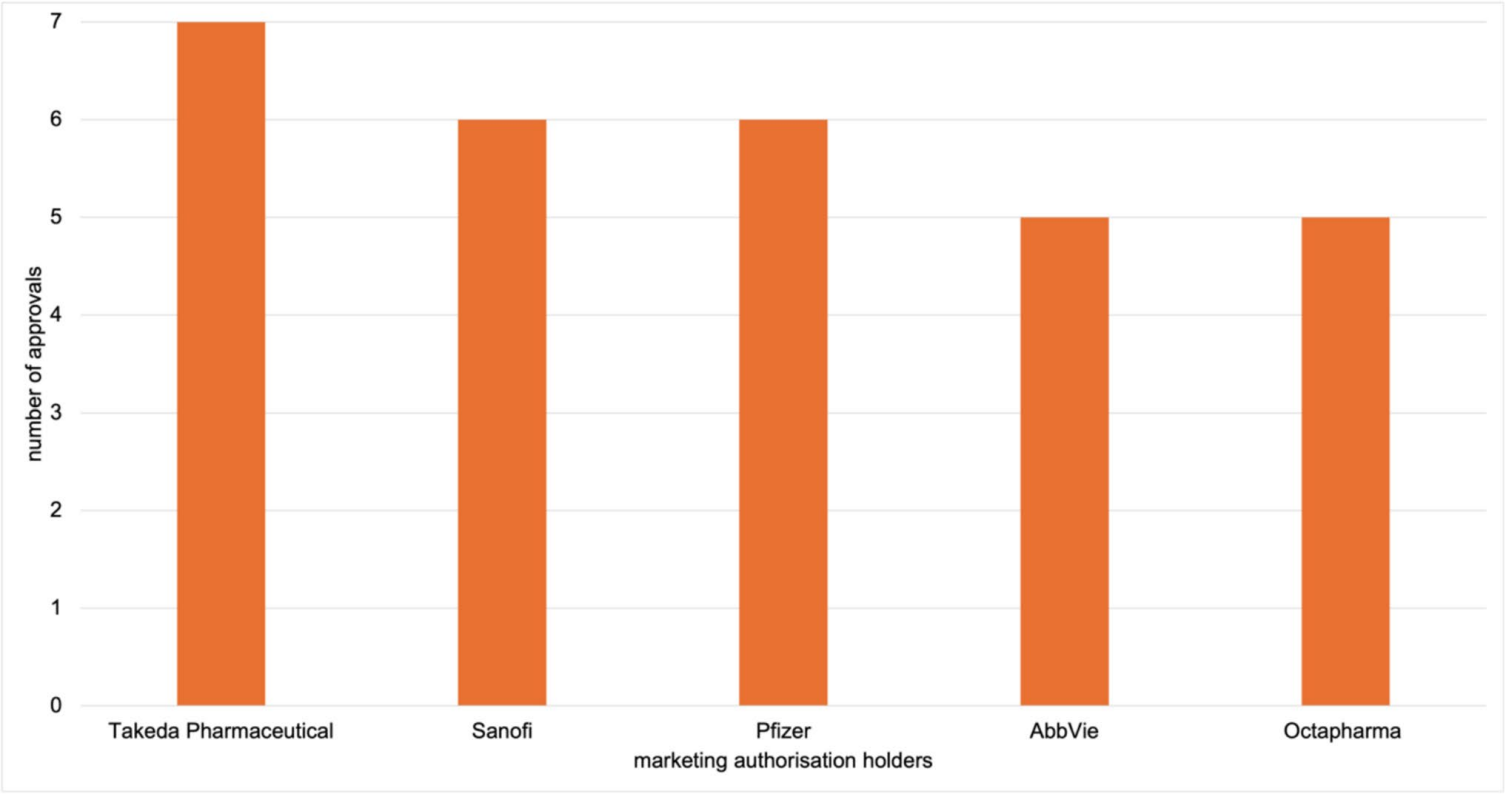
Top 5 marketing authorisation holders of FDA exclusive novel drug approvals from 2013 to 2023, shown in a bar chart

The MAHs of exclusive drug approvals were analysed. Overall, the 42 EMA-exclusive authorisations are held by 34 MAHs. In contrast, 185 FDA-exclusive approvals are held by 134 companies. Figures 9 and 10 show the top 5 MAHs of exclusive drug approvals for each agency. For the EMA, AstraZeneca, Chiesi Farmaceutici, and Johnson and Johnson each hold 3 marketing authorisations, followed by Takeda Pharmaceutical and PTC Therapeutics with 2 each. AstraZeneca, Johnson and Johnson, and Takeda Pharmaceutical also appear among the top 15 MAHs for overall approvals (Table 4). In contrast, Chiesi Farmaceutici and PTC Therapeutics are not represented in the overall top 15 MAHs but are specific to EMA-exclusive approvals.

For the FDA, Takeda Pharmaceutical holds 7 exclusive approvals, followed by Sanofi and Pfizer with 6 each, and AbbVie and Octapharma with 5 each. Takeda Pharmaceutical, Sanofi, Pfizer, and AbbVie also rank among the top 15 MAHs for overall approvals (Table 4). Octapharma, however, appears only in the context of FDA-exclusive approvals. Takeda Pharmaceutical is the only company represented among the most common MAHs for both overall and exclusive approvals by both agencies. To complement the absolute values, Supplementary Figs. 14 and 15 show the data as relative percentages.

**Fig. 11:**
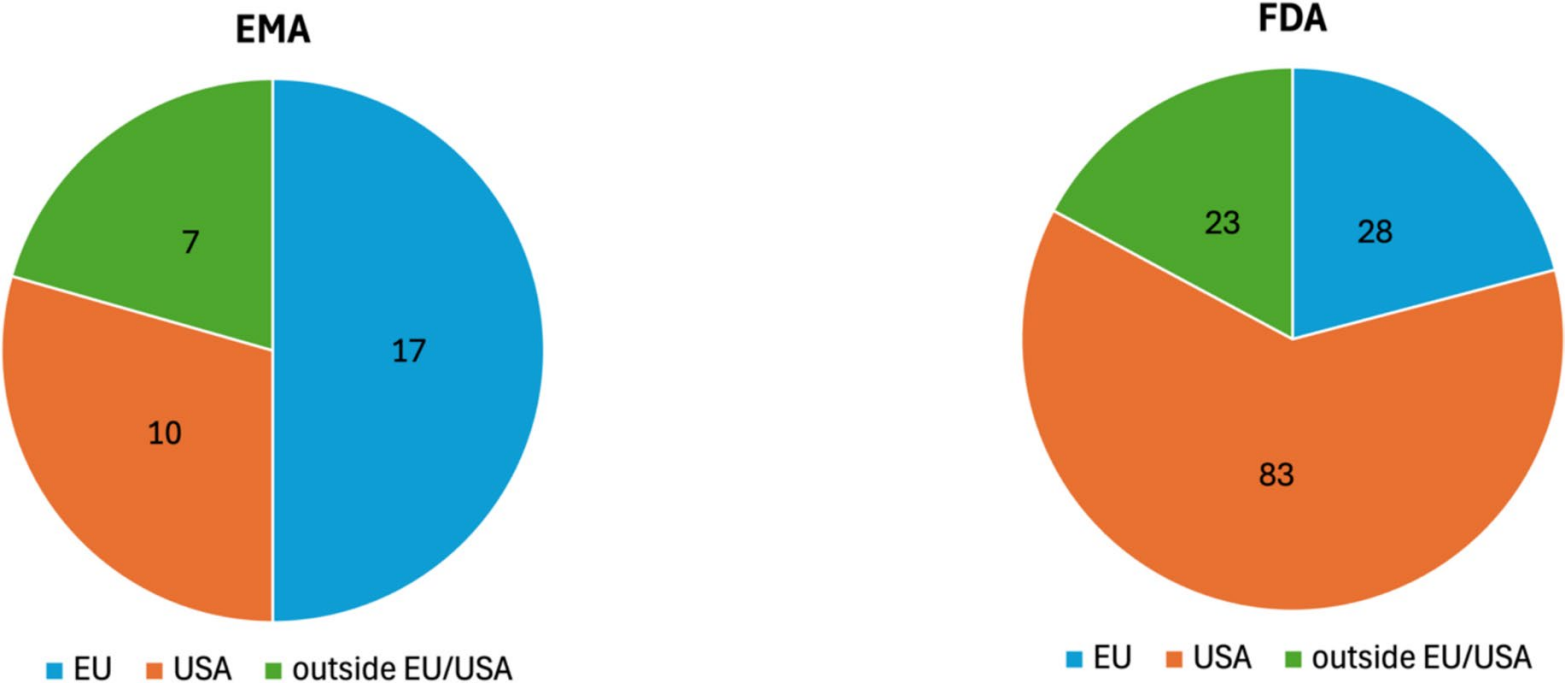
Distribution of company headquarters of marketing authorisation holders for exclusive drug approvals from 2013 to 2023, based on the total number of authorisation holders, shown in two pie charts

Figure 11 shows the distribution of MAH headquarters for agency-exclusive approvals. Among the 34 EMA MAHs, 17 are based in the EU, 10 in the USA, and 7 in other regions.

In contrast, of 134 FDA MAHs, 83 are based in the USA, 28 in the EU, and 23 elsewhere. These findings align with previously observed patterns in overall drug approvals: while both agencies prioritise companies from their own region, this regional focus appears stronger for the FDA. However, the proportion of EU-based MAHs among EMA-exclusive approvals is significantly higher than in overall EMA approvals (Fig. 8). Consequently, the share of US-based companies is smaller among EMA-exclusive approvals compared to overall EMA approvals. Supplementary Fig. 16 presents these data as relative percentages.

### Differences in approval timing of joint approvals

**Fig. 12:**
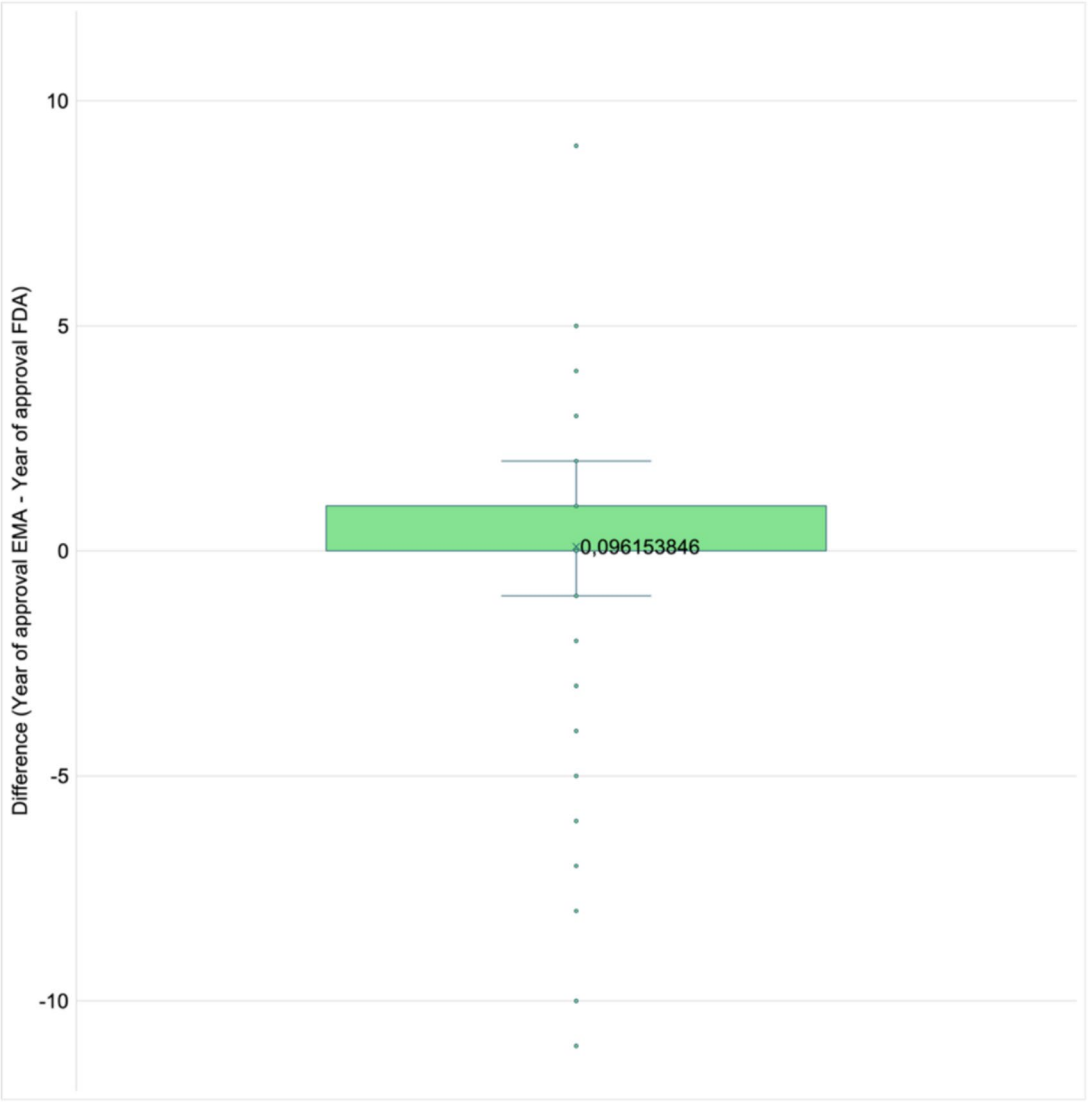
Difference in approval years between the EMA and FDA for joint drug approvals, shown in a box plot. The box represents the interquartile range (IQR); whiskers extend to 1.5 times the IQR below the first quartile (Q1) and above the third quartile (Q3). Dots outside the box highlight outliers beyond this range. The cross marks the mean difference

Figure 12 shows approval time differences between the EMA and the FDA for joint drug approvals, calculated by subtracting the FDA approval year from the EMA approval year. A positive value indicates that the EMA approved the drug later than the FDA, while a negative value indicates earlier approval by the EMA. The median difference of 0.096 years is marked by a cross within the box. The box represents the interquartile range from 0 to 1 year. The whiskers span from − 1 to 2. Outliers, shown as individual points, range from − 11 to + 9. On average, the EMA grants approval slightly later than the FDA, by approximately 0.096 years (or about 1.15 months). Most differences fall within a range from the EMA approving drugs 1 year earlier to 2 years later than the FDA. A few extreme outliers range from the EMA approving a drug 11 years earlier to 9 years later.

## Discussion

### Differences in approval numbers

During the observed period, the FDA approved significantly more novel drugs than the EMA. Between 2013 and 2023, the FDA authorised a total of 583 NMEs, including 185 FDA-exclusive approvals. In contrast, the EMA approved 424 NASs, of which 42 were EMA-exclusive. This difference is largely due to structural differences in the regulatory systems. While the FDA functions as the single national authority for drug regulation in the USA, the EMA is responsible only for centrally authorised medicines. However, there are three additional approval pathways: national authorisation, decentralised authorisation, and the mutual-recognition procedure (https://www.ema.europa.eu/en/about-us/what-we-do/authorisation-medicines?utm_source=chatgpt.com, last accessed March 31, 2025). As a result, certain drugs may not appear in the Annual Report if they were authorised via national procedures by individual EU member states.

Another important factor is market strategy. MAHs do not always submit applications to both agencies simultaneously and may strategically prioritise certain markets. Lythgoe et al. (2022) found that 72% of marketing applications for cancer treatments from 2010 to 2019 were submitted to the FDA before the EMA. The study attributes this in part to the larger pharmaceutical market and higher pricing levels for new oncology therapeutics in the USA.

The notably high number of FDA-exclusive approvals in 2023 (Fig. 3) may also reflect the end of the observation period, as some of these drugs may have been authorised by the EMA afterwards or are still under review.

### Financial frameworks and decision-making processes

Table 5 provides an overview of the financial frameworks and decision-making processes of the EMA and FDA. The FDA is funded through a combination of public funds and industry fees. In 2024, industry fees accounted for 69.2% of the FDA’s human drug regulatory budget (US Food and Drug Administration 2024a). These fees are renegotiated every 5 years as part of the Prescription Drug User Fee Act (PDUFA), an agreement between the FDA and the pharmaceutical industry. PDUFA was introduced specifically to accelerate review times and defines the FDA’s performance goals (https://www.fda.gov/news-events/congressional-testimony/fda-user-fee-reauthorization-ensuring-safe-and-effective-drugs-and-biologics-02032022, last accessed March 19, 2025). This system creates economic pressure on the FDA to make quick decisions and may increase its dependence on the pharmaceutical industry. Previous studies suggest that the recurring renegotiation of PDUFA has increased industry influence over regulatory processes. For example, accelerated approval pathways have been expanded, and scientific standards have been lowered, leading to acceptance of less robust clinical data (Mitchell et al. 2022).

**Table 5:** Overview of financial frameworks and decision-making of the EMA and the FDA (based on data discussed in the main text)

	**FDA**	**EMA**
Funding	• Budget authority (public funding) • Industry fees (PDUFA) o To accelerate the approval process o 69.2% of the human drug regulatory budget in 2024	• Contributions from EU Member States • Grants from the European Commission • Industry fees o For various phases of the authorization and monitoring process o 91.5% of the budget in 2025 (projected)
Decision-making pressure	• Strong economic pressure to make quick decisions	• Lower economic pressure
Review timelines	• Performance goals as part of the PDUFA agreements • Renegotiated every 5 years between the FDA and the pharmaceutical industry • PDUFA fees enable faster review times	• Legally defined deadlines
Focus	• Fast and efficient processes	• Long-term safety

The EMA is funded by contributions from EU member states, grants from the European Commission, and primarily by industry fees. These fees are expected to account for 91.5% of its total budget in 2025 (https://www.ema.europa.eu/en/about-us/how-we-work/governance-reporting/funding, last accessed March 19, 2025). Unlike the FDA, EMA fees are not linked to faster review times, as the agency operates under legally defined deadlines and charges fees for various phases of the authorisation and monitoring process (European Union 2024; European Medicines Agency 2019). Consequently, the EMA is under less economic pressure to accelerate approval decisions and may focus on long-term safety rather than speed and efficiency.

### Exclusive drug approvals

While differences in financial frameworks and decision-making provide insight into general regulatory processes, more distinct discrepancies emerge when analysing agency-exclusive drug approvals. These cases may reflect regulatory priorities and strategic decisions. Although there are various reasons why a drug might be authorised by only one agency, such as differences in submission timing or the absence of marketing application, this analysis focuses specifically on drugs rejected by one agency following regulatory review. Accordingly, Table 6 shows a selection of examples where an approval by one agency directly contrasts with a negative evaluation from the other, offering insight into differing regulatory decisions. These examples were selected to show a variety of rejection reasons to highlight the range of factors that may lead to different outcomes.

**Table 6:** Selection of agency-exclusive drug approvals from 2013 to 2023

Active substance	Commercial name	Mechanism of action/molecular target	Condition treated	Year of approval	Marketing auth. holder	Approved by	Rejected by	Reasons for rejection
Roxadustat	Evrenzo	Prolyl hydroxylase inhibitor	Anaemia caused by chronic kidney failure	2021	Astellas Pharma	EMA^a^	FDA^b^	Concerns about the safety profile (thrombo-embolic risk above standard of care) and data manipulation of the Phase III study
Bulevirtide	Hepcludex	Sodium taurocholate co-transporting polypeptide antagonist	Chronic hepatitis delta virus (HDV) infection	2020	Gilead Science	EMA^c^	FDA^d^	Concerns about manufacturing and delivery
Filgotinib	Jyseleca	Janus kinase (JAK) inhibitor	Rheumatoid arthritis; ulcerative colitis	2020	Gilead Science	EMA^c^	FDA^e^	Concerns about the drug’s safety profile particularly at higher doses (200 mg dose)
Ataluren	Translarna	Gene-/cell therapy	Duchenne muscular dystrophy	2014	PTC Therapeutics	EMA^f^	FDA^g^	Concerns about insufficient evidence for its efficacy
Volanesorsen	Waylivra	ApoC-III formation inhibition	Familial chylomicronaemia syndrome (FCS)	2019	Ionis Pharmaceuticals	EMA^h^	FDA^i^	Concerns about serious side effects (low blood platelet counts)
Aducanumab	Aduhelm	Anti-amyloid monoclonal antibody	Alzheimer’s disease	2021	Biogen	FDA^j^	EMA^k^	Concerns about the safety profile (risk of amyloid-related imaging abnormalities (ARIA))
Eteplirsen	Exondys 51	Exon skipping therapy	Duchenne muscular dystrophy (DMD)	2016	Sarepta Therapeutics	FDA^l^	EMA^m^	Concerns about the robustness of data from clinical trials and insufficient clinical evidence
Mipomersen	Kynamro	Gene-/cell therapy	Homozygous familial hypercholesterolemia	2013	Sanofi	FDA^n^	EMA^o^	Concerns about the safety profile (potential liver toxicity)
Lecanemab	Leqembi	Anti-amyloid monoclonal antibody	Alzheimer’s disease	2023	Eisai	FDA^p^	EMA^q^	Concerns about the safety profile (risk of amyloid-related imaging abnormalities (ARIA))

^a^European Medicines Agency (2022)

^b^https://www.astrazeneca.com/media-centre/press-releases/2021/update-on-us-review-of-roxadustat.html#, last accessed March 25, 2025

^c^European Medicines Agency (2021d)

^d^https://www.gilead.com/company/company-statements/2022/gilead-receives-complete-response-letter-from-us-fda-for-bulevirtide-for-the-treatment-of-adults-with-hepatitis-delta-virus, last accessed March 26, 2025

^e^https://www.gilead.com/news/news-details/2020/gilead-receives-complete-response-letter-for-filgotinib-for-the-treatment-of-moderately-to-severely-active-rheumatoid-arthritis?utm_source=chatgpt.com, last accessed March 26, 2025

^f^European Medicines Agency (2015b)

^g^https://ir.ptcbio.com/news-releases/news-release-details/ptc-receives-refuse-file-letter-fda-translarnatm-ataluren?utm_source=chatgpt.com, last accessed March 25, 2025

^h^European Medicines Agency (2020)

^I^https://ir.ionis.com/news-releases/news-release-details/akcea-and-ionis-receive-complete-response-letter-waylivra-fda?utm_source=chatgpt.com, last accessed March 25, 2025

^j^US Food and Drug Administration (2022b)

^k^European Medicines Agency (2021c)

^l^US Food and Drug Administration (2017)

^m^European Medicines Agency (2018)

^n^US Food and Drug Administration (2014)

^o^European Medicines Agency (2013)

^p^US Food and Drug Administration (2024b)

^q^At the time of analysis, Leqembi had been rejected by the EMA. However, in November 2024, the drug was approved following re-examination (European Medicines Agency 2025)

Evrenzo (Roxadustat), for instance, was approved by the EMA in 2021 for the treatment of symptomatic anaemia in patients with chronic kidney failure (European Medicines Agency 2022). However, the FDA rejected the application due to safety concerns and requested additional clinical trials (https://www.astrazeneca.com/media-centre/press-releases/2021/update-on-us-review-of-roxadustat.html#, last accessed March 25, 2025). Furthermore, FibroGen, the company pursuing US approval in collaboration with AstraZeneca, applied post hoc changes to its phase III trial analysis. This led to more favourable results, particularly in the dialysis cohort (https://investor.fibrogen.com/news-releases/news-release-details/fibrogen-provides-additional-information-roxadustat, last accessed March 25, 2025).

As of March 2025, no additional clinical trials have been initiated. In 2024, AstraZeneca and FibroGen ended their collaboration on Roxadustat, with AstraZeneca returning the US rights to FibroGen (https://investor.fibrogen.com/news-releases/news-release-details/fibrogen-regains-all-rights-roxadustat-astrazeneca-united-states?utm_source=chatgpt.com, last accessed March 25, 2025).

While no official statement rules out future clinical trials and approval applications, the decision implies a strategic withdrawal from pursuing FDA approval for now. In contrast, the EMA granted approval for Evrenzo in 2021. Although specific risks such as thrombotic vascular events were identified, the EMA concluded that the overall benefits-risks balance was positive. The MAH was required to follow a risk management plan and submit periodic safety updates (European Medicines Agency 2021b).

This scenario can also occur in the opposite direction: Aduhelm (Aducanumab) was approved by the FDA in 2021 for the treatment of Alzheimer’s disease (US Food and Drug Administration 2022b). However, the EMA had safety concerns, particularly regarding amyloid-related imaging abnormalities (ARIA). Furthermore, Biogen had submitted two phase III trials, both of which were terminated early due to futility. Consequently, further evaluation was limited to post hoc analyses, which presented inconsistent results and did not align with the original hypothesis. The EMA also found insufficient evidence for the correlation between beta-amyloid reduction and clinical improvement. As a result, the application was rejected, with the EMA concluding that the benefits did not outweigh the risks (European Medicines Agency 2021c). The FDA interpreted the data differently. The observed reduction in amyloid plaques was accepted as a surrogate endpoint, and Aduhelm was granted Accelerated Approval. This approval pathway allows earlier access to drugs for critical health conditions with limited treatment options based on surrogate or intermediate clinical endpoints. Under this programme, the MAH is required to confirm clinical benefit in post-marketing (phase IV) trials. Given the high unmet need in Alzheimer’s disease, the FDA granted Accelerated Approval for Aduhelm in 2021 despite uncertainty about clinical efficacy (https://www.fda.gov/drugs/our-perspective/fdas-decision-approve-new-treatment-alzheimers-disease, last accessed March 26, 2025).

Another example is Hepcludex (Bulevirtide), approved by the EMA in 2020 for treatment of chronic hepatitis delta virus infection (European Medicines Agency 2021d). The FDA rejected the application due to manufacturing and supply concerns. Notably, no additional data on safety and efficacy was requested, suggesting that these aspects did not raise concern (https://www.gilead.com/company/company-statements/2022/gilead-receives-complete-response-letter-from-us-fda-for-bulevirtide-for-the-treatment-of-adults-with-hepatitis-delta-virus, last accessed March 26, 2025).

These examples show that there are various reasons causing discrepancies in approval decisions. In most cases, rejections result from concerns about safety or efficacy. Kashoki et al. (2020) found that divergent approval decisions, which mostly occur for oncology and haematology drugs, were mostly based on differing interpretations of the same data regarding efficacy or on the submission of different clinical data. The second factor mentioned is due to a later submission to the EMA, which often results in additional and more mature clinical data being available for EMA assessment (Kashoki et al. 2020). Lythgoe et al. (2022) confirmed this trend for cancer therapy drugs: 72% of marketing applications from 2010 to 2019 were submitted to the FDA before the EMA.

Organisational differences between the agencies also play a role. The EMA’s Committee for Medicinal Products for Human Use (CHMP) includes representatives from all EU member states (https://www.ema.europa.eu/en/committees/committee-medicinal-products-human-use-chmp/chmp-members, last accessed March 28, 2025). This broad representation may lead to consensus-driven decision-making and a more cautious approach.

In contrast, the FDA is a federal agency (https://www.fda.gov/about-fda/fda-organization, last accessed March 28, 2025), which may allow more internally consistent decisions.

Alternative approval pathways also contribute to divergent outcomes. As mentioned before, the FDA’s Accelerated Approval programme allows authorisation based on less robust data to drugs addressing an unmet medical need (https://www.fda.gov/drugs/nda-and-bla-approvals/accelerated-approval-program, last accessed March 28, 2025). The EMA’s Conditional Marketing Authorisation (CMA) offers a similar option (https://www.ema.europa.eu/en/human-regulatory-overview/marketing-authorisation/conditional-marketing-authorisation, last accessed March 28, 2025). Xie et al. (2023) compared novel oncology drugs approved under these pathways and found that the FDA uses them more frequently and is more willing to accept uncertainty. The EMA, however, tends to require more extensive post-marketing studies addressing both efficacy and safety (Xie et al. 2023).

The case of Aduhelm shows how even minor differences in otherwise comparable approval pathways can result in different outcomes. While the FDA accepted amyloid reduction as surrogate endpoint and granted Accelerated Approval, the EMA rejected the application, finding the surrogate not sufficiently validated to demonstrate clinical benefit (European Medicines Agency 2021c); https://www.fda.gov/drugs/our-perspective/fdas-decision-approve-new-treatment-alzheimers-disease, last accessed March 26, 2025).

### Therapeutic priorities

The analysis of overall approvals shows a strong focus by both agencies on malignant disease. This reflects global health priorities and ongoing innovation in oncology (Huber and Huber 2019). Other major areas, such as haematology/haemostaseology, neurology, and infectious diseases, are similarly represented. An exception is the category COVID-19: the EMA approved significantly more drugs in this area, which may suggest differing approaches to pandemic response and public health coordination. Overall, the therapeutic focus appears broadly consistent, suggesting that, despite differences in regulatory frameworks and approval processes, both agencies are largely aligned in their priorities. This is supported by findings from Kashoki et al. (2020), who found that between 2014 and 2016, the initial marketing authorisation decisions of the EMA and FDA were concordant in 91% of cases.

However, this alignment diminishes when analysing agency-exclusive approvals. Here, distinct differences emerge: the EMA places stronger emphasis on COVID-19 therapeutics, suggesting a broader public health response at the EU level. In contrast, the FDA shows a broader distribution across more therapeutic areas. Some categories, such as diagnostic agents and psychiatry, appear exclusively in the FDA approvals, possibly reflecting differences in market dynamics or regulatory flexibility.

Overall, the agencies are broadly aligned in major therapeutic areas but differ in how they address specialised or niche therapeutic areas.

An analysis of mechanisms of action provides further pharmacological perspective on how therapeutic priorities are addressed. Both agencies show a shared focus on enzyme inhibitors, followed by antibodies and replacement/substitution/transplantation therapies. However, notable differences appear among less common mechanisms and exclusive approvals. FDA-exclusive drugs cover a broader range of mechanisms, including categories not represented in EMA-exclusive approvals, such as diagnostic/contrast agents and RNA/DNA modifiers. In contrast, vaccines and gene-/cell therapy are dominantly represented among EMA-exclusive approvals.

These differences reflect varying strategic priorities and public health needs. In addition, the FDA shows a higher number of approvals with unclear mechanisms, suggesting greater acceptance of novel or incompletely understood therapeutic approaches. This aligns with the FDAs’ long experience with accelerated approval pathways and a generally greater acceptance of uncertainty in benefit-risk assessments (Xie et al. 2023).

These findings are consistent with the annual review articles on new FDA drug approvals by Kayki-Mutlu and Michel (2021), Kayki-Mutlu et al. (2022, 2023, 2024), and Aksoyalp et al. (2025). They report continued high use of accelerated approval pathways (Fast Track, Breakthrough Therapy, Accelerated Approval, Priority Review) and a growing share of RNA- and gene-based therapies. In 2023, for example, 41% of approvals received Fast Track designation, a 9% increase compared to previous years. In 2024, this figure rose to over 50%. Oncology consistently emerged as the leading therapeutic area, confirming the trends identified in this study. The reviews show an increase of First-in-Class drugs over the years, highlighting the agency’s innovation-driven approach. Furthermore, the consistently high share of orphan drug approvals, accounting for over 50% in recent years, helps explain the broader diversity of mechanisms and therapeutic areas seen in FDA approvals. This reflects a strategic interest in targeting rare diseases. The decreasing number of small molecules and increase of biologics and RNA- and gene-based therapies further indicate a shift in innovation strategies, aligning with this study’s finding of a more exploratory pharmacological profile at the FDA. Overall, while the FDA and EMA align in major pharmacological strategies, their approaches to innovation and regulatory risk differ.

Differences in COVID-19-related approvals and the higher number of EMA-approved vaccines can partly be attributed to the use of alternative approval pathways. While the EMA primarily used CMAs to enable earlier access to urgently needed drugs, the FDA primarily granted EUAs. Like EUAs, CMAs are granted under exceptional circumstances during public health emergencies based on less comprehensive data. However, unlike EUAs, CMAs are considered formal marketing authorisations and are thus included in the EMA’s Annual Reports as NASs. In contrast, EUAs are considered provisional authorisations and are not included in the FDA’s New Drug Therapy Approvals Reports. Therefore, they are not part of this analysis (https://www.ema.europa.eu/en/human-regulatory-overview/marketing-authorisation/conditional-marketing-authorisation?utm_source=chatgpt.com; https://www.fda.gov/emergency-preparedness-and-response/mcm-legal-regulatory-and-policy-framework/emergency-use-authorization?utm_source=chatgpt.com; https://www.fda.gov/drugs/development-approval-process-drugs/novel-drug-approvals-fda, all last accessed April 1, 2025; European Medicines Agency 2023).

### Marketing authorisation holders and geographical factors

The FDA and EMA share the same top 3 MAHs: Pfizer, Novartis, and AstraZeneca. Their similar representation suggests a global marketing approach by these companies (https://www.pfizer.com/about/responsibility/global-impact; https://www.novartis.com/news/media-releases/novartis-unveils-new-focused-strategy-underpinned-eight-potential-multi-billion-dollar-peak-sales-brands-deep-pipeline-meet-management-event?utm_source=chatgpt.com; https://www.astrazeneca.com/our-company.html?%C2%A0=&utm_source=chatgpt.com all last accessed April 2, 2025). However, FDA approvals are distributed across a greater number of MAHs, indicating a more diverse company landscape. The geographical distribution of MAHs reveals that both agencies tend to approve more drugs from domestic companies. US-based companies are more frequently represented among EMA approvals than EU-based companies are among FDA approvals. This asymmetry may reflect a stronger global presence of US-based companies and a stronger domestically oriented focus of the FDA. MAHs based outside the EU and USA account for the smallest share of approvals for both agencies. Further differences emerge when examining agency-exclusive approvals. The most frequently represented MAHs differ from those seen in overall approvals. FDA-exclusive approvals continue to be distributed across a broader range of MAHs.

Notably, Takeda Pharmaceuticals stands out as the only company represented among the top MAHs in all categories: overall approvals, as well as EMA- and FDA-exclusive approvals. This suggests strong global positioning and regulatory adaptability (https://www.takeda.com/newsroom/newsreleases/2021/takedas-growth-and-emerging-markets-business-unit-aims-to-deliver--double-digit-revenue-growth-over-next-decade/, last accessed April 2, 2025).

In terms of geography, slight shifts are observed when focusing on exclusive approvals. For the EMA, the regional focus becomes more pronounced, as EMA-exclusive approvals show a stronger emphasis on EU-based companies. In contrast, the FDA maintains a consistent US-centred focus across both overall and exclusive approvals. All in all, the representation of companies in drug approvals reflects regulatory and market dynamics. The FDA’s wider range of MAHs may indicate a more diverse and innovation-oriented approach (https://www.fda.gov/regulatory-information/selected-amendments-fdc-act/21st-century-cures-act, last accessed April 2, 2025). At the same time, the FDA demonstrates a consistent preference for domestic companies. In comparison, EMA approvals appear more centralised and, in the case of EMA-exclusive drugs, more regionally concentrated than observed for overall approvals.

### Differences in approval timing

Downing et al. (2012) analysed novel drug approvals by the FDA, EMA, and Health Canada from 2001 to 2010, focusing on regulatory review times. They found that the FDA had the shortest total review time and was first to approve novel drugs in most cases. On average, the FDA authorised new drugs 90 to 100 days (about 3 months) earlier than the EMA. A more recent study by Lythgoe et al. (2022) examined oncology therapeutics approved between 2010 and 2019. They found that 95% were approved earlier by the FDA, with an average delay of 241 days (about 8 months) in the EU. These data fit to the data of our present study showing a modest delay of EMA relative to the FDA.

### Global harmonisation and regulatory cooperation

Despite the differences identified in this study, both agencies acknowledge the value of international harmonisation to align regulatory processes and decisions. Programmes such as Parallel Scientific Advice (PSA) aim to enhance exchange between the FDA and EMA during the development phase of new drugs. They aim to reduce regulatory differences and prevent unnecessary duplication of studies or conflicting testing requirements (European Medicines Agency and US Food and Drug Administration 2021). The FDA and the European Commission, which grant the final, legally binding marketing authorisation in the EU, are founding regulatory members of the International Council for Harmonisation of Technical Requirements for Pharmaceuticals for Human Use (ICH) (https://www.ich.org/page/members-observers, last accessed March 31, 2025). The ICH promotes collaboration between regulatory authorities and the pharmaceutical industry to advance the global harmonisation of technical requirements. This includes supporting the mutual acceptance of data and the implementing of internationally harmonised scientific standards (https://www.ich.org/page/mission, last accessed March 31, 2025). Furthermore, the FDA initiated Project Orbis, an initiative to enhance simultaneous and faster approval of oncology drugs across multiple countries (https://www.fda.gov/about-fda/oncology-center-excellence/project-orbis?utm_source=chatgpt.com, last accessed March 31, 2025). While the EMA is not an active participant, harmonisation efforts remain evident, as it joined Project Orbis as an observer (European Medicines Agency 2024). These examples demonstrate that both agencies recognise the importance of global harmonisation.

At the same time, they reveal existing limitations and the continued need for broader, more inclusive collaboration, especially for therapies addressing global unmet medical needs.

### Limitations of the study

This study covers a fixed period from 2013 to 2023. As the FDA and EMA continue to approve new drugs, the results represent a snapshot in time and may evolve as additional authorisations are granted. Part of the study period overlaps with the COVID-19 pandemic, which may have influenced regulatory priorities and decision-making processes. In this analysis, only approval years were considered. This does not necessarily reflect the actual point in time at which a drug becomes available to patients. For example, in the EU, final marketing authorisation is granted by the European Commission following a recommendation from the EMA. Since submission dates were not analysed, differences in approval timelines may reflect not only regulatory speed but also strategic submission decisions made by pharmaceutical companies. In this context, it is also important to note that agency-exclusive approvals are influenced by both regulatory and company-level strategies. The FDA and EMA differ in how they classify and report accelerated or alternative approval pathways. Therefore, some products may have received authorisation but were not included in the respective reports and were therefore excluded from this analysis. The same applies to drugs approved in the EU via decentralised or national procedures. Since the EMA is responsible only for centrally authorised products, such cases were not examined. Consequently, some drugs categorised as FDA-exclusive may still be available to patients in the EU.

### Conclusions and future studies

The study highlights differences and similarities between the FDA and EMA in their drug approval decisions from 2013 to 2023. While the agencies show general alignment in therapeutic focus and approval timelines, notable discrepancies remain approval numbers and regulatory approaches.

The FDA authorised more novel drugs overall, partly due to regulatory structures and the EMA’s limited responsibility for the centralised procedure. Additionally, the FDA’s greater speed and flexibility, contrasted with the EMA’s focus on long-term safety, result from differing financial frameworks and decision-making processes.

The analysis of agency-exclusive approvals shows different interpretations of the same clinical data and varying acceptance of surrogate endpoints. The use of accelerated approval pathways further contributes to these discrepancies. Additionally, MAHs pursue independent market strategies, including submission timing and regional prioritisation, which also influence observed differences.

In addition to MAH-driven differences, regional patterns may also reflect agency-level preferences. Both the EMA and FDA tend to favour domestic companies, with this preference being more consistent for the FDA. For the EMA, this is particularly evident in exclusive approvals. In addition, the FDA collaborates with a broader range of MAHs, indicating a more diverse and globally engaged industry landscape. This highlights the complex interplay between regulatory frameworks, market orientation, and submission strategies at the company level.

When examining therapeutic areas and mechanisms of action, shared priorities become evident. Both agencies show alignment in major fields, such as oncology and infectious diseases and focus on similar pharmacological strategies, including enzyme inhibitors and antibodies. However, the FDA approves a broader range of drugs, including a higher number of drugs with unclear mechanisms.

In contrast, the EMA focuses more on public health priorities, such as COVID-19 and vaccine approvals, especially among its exclusive approvals. This difference is partially structural, as the FDA’s EUAs are not included in the New Drug Therapy Approvals Reports. Altogether, these findings suggest a more exploratory approach at the FDA and a stronger public health orientation at the EMA. Despite these differences, both agencies demonstrate a commitment to harmonisation. Initiatives such as PSA, ICH, and Project Orbis reflect ongoing efforts toward greater regulatory cooperation. However, limitations remain, particularly in the context of urgent, unmet medical needs.

In conclusion, while the EMA and FDA increasingly align on overall priorities, strategic and structural differences continue to shape divergent regulatory outcomes.

Future studies could include drug authorisations granted through national or decentralised procedures in the EU. This would provide a more comprehensive overview of drug availability and reveal regional discrepancies in patient access. Furthermore, the effectiveness of harmonisation efforts could be examined, both in terms of patient access to therapeutics and in reducing complexity for companies submitting applications to multiple agencies. A systematic analysis of how companies adapt to different regulatory requirements, especially large multinational companies, could provide insight into how international processes, submission strategies and data presentation change to meet agency-specific expectations. Further research might also examine whether faster regulatory approval translates into improved patient outcomes, offering a public health perspective. So far, research has primarily focused on approval speed and access rather than broader public health impact of regulatory differences.

### Take-home messages

- The FDA authorises more novel drugs than the EMA, primarily due to differences in regulatory processes and market dynamics.
- The FDA tends to use faster and more flexible approval processes than the EMA.
- The FDA shows a broader acceptance of therapeutics with less well-defined pharmacological profiles, indicating greater tolerance for uncertainty and risk.
- Agency-exclusive approvals often indicate differing interpretations of clinical data and variations in regulatory risk tolerance.
- Therapeutic focus of the agencies is largely aligned in major areas. However, agency-exclusive approvals reveal notable differences in less-represented areas and pandemic-related therapies.
- The distribution of MAHs across approvals reflects both marketing strategies and regional regulatory patterns.
- Both agencies make efforts to achieve harmonisation. However, further collaboration is needed to address remaining regulatory differences.

## Supplementary Information

Below is the link to the electronic supplementary material.

ESM 1 (DOCX 1.74 MB)

## Data Availability

All source data for this study are available upon reasonable request.

## Abbreviations

CHMP: Committee for Medicinal Products for Human Use
CMA: Conditional Marketing Authorisation
EU: European Union
EUA: Emergency Use Authorisations
EMA: European Medicines Agency
FDA: US Food and Drug Administration
ICH: International Council for Harmonisation of Technical Requirements for Pharmaceuticals for Human Use
MAH: Marketing authorisation holder
MSD: Merck Sharp and Dohme
NAS: New active substance
NCE: New chemical entity
NME: New molecular entity
PDUFA: Prescription Drug User Fee Act
PSA: Parallel scientific advice
USA: United States of America
