# Supplementary material for: Comparison of drug approvals of the FDA and EMA between 2013 and 2023
Source: Naunyn Schmiedebergs Arch Pharmacol. 2025 Jul 3;399(1):279–99. doi: 10.1007/s00210-025-04412-4 (PMC12894151; doi:10.1007/s00210-025-04412-4)
Supplement: Supplementary file 1 — (DOCX 1.74 MB) [file 210_2025_4412_MOESM1_ESM.docx]

**Comparison of drug approvals in Europe and the USA: What are the differences between the FDA and the EMA?**

**Franziska Lau and Roland Seifert**

**Supplementary information**

**
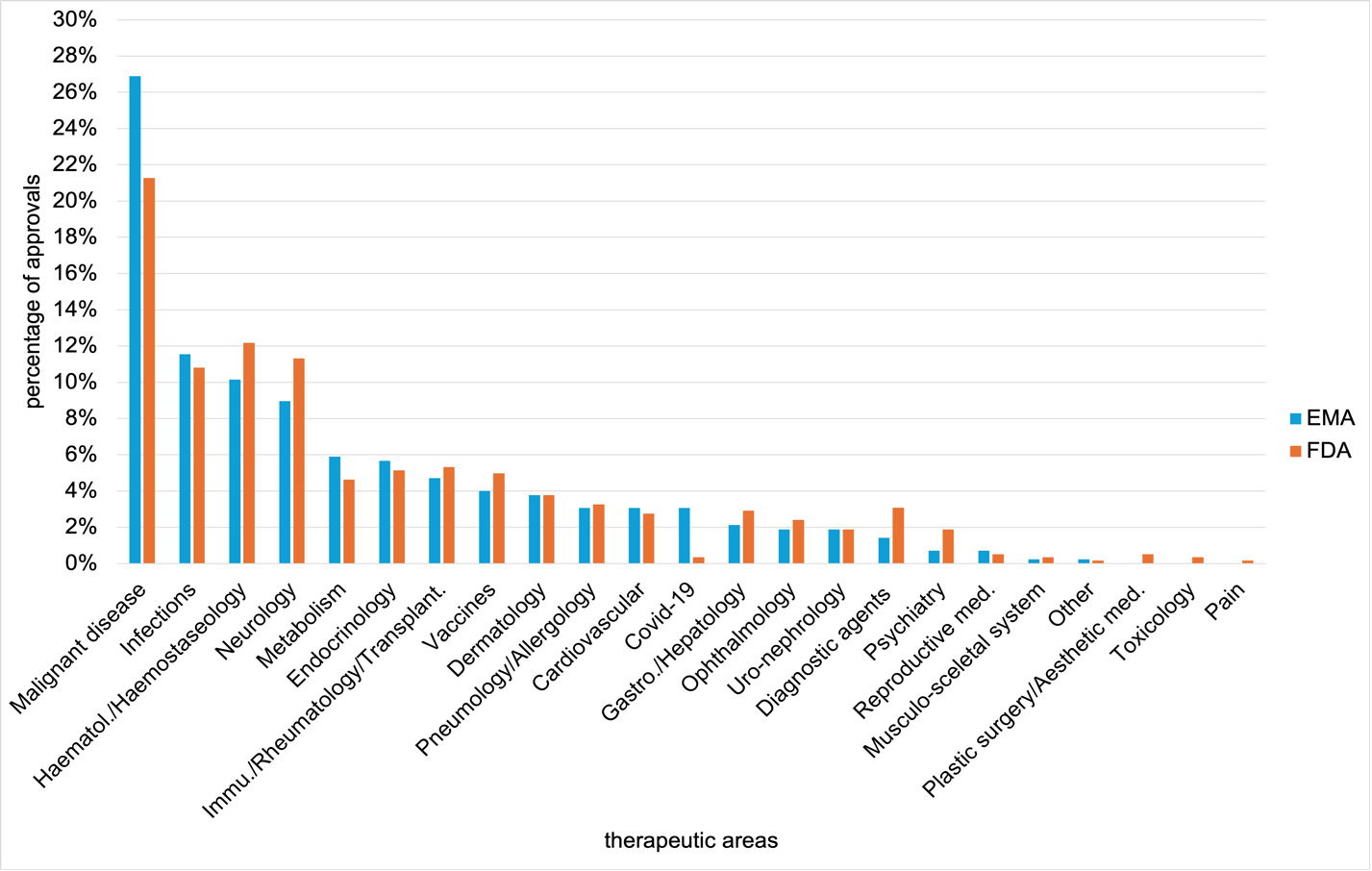

Fig. S1** Percentage distribution of EMA and FDA novel drug approvals across therapeutic areas from 2013-2023, shown in a bar chart

**
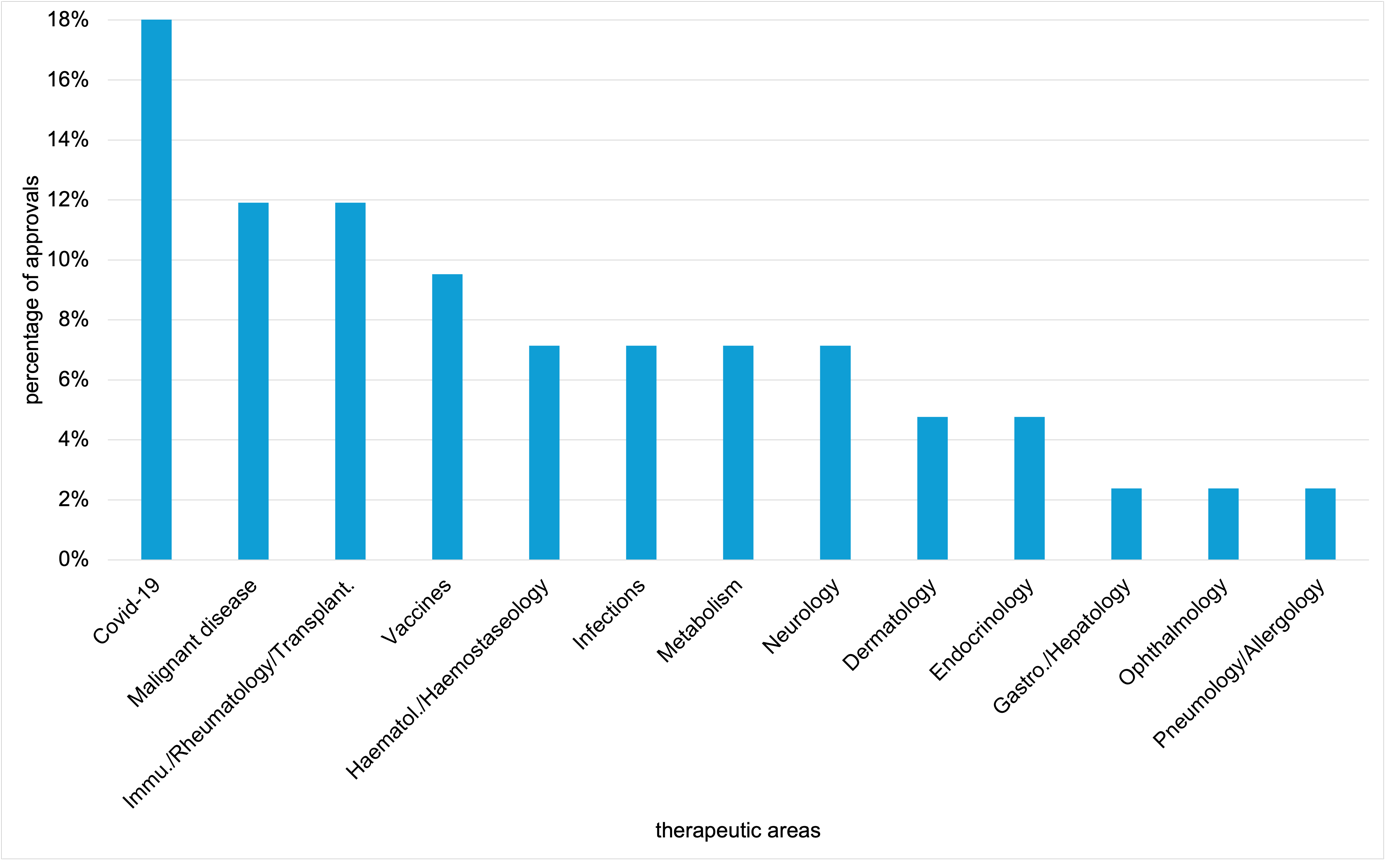
Fig. S2** Percentage distribution of EMA novel drug approvals by therapeutic areas from 2013 to 2023, shown in a bar chart

**
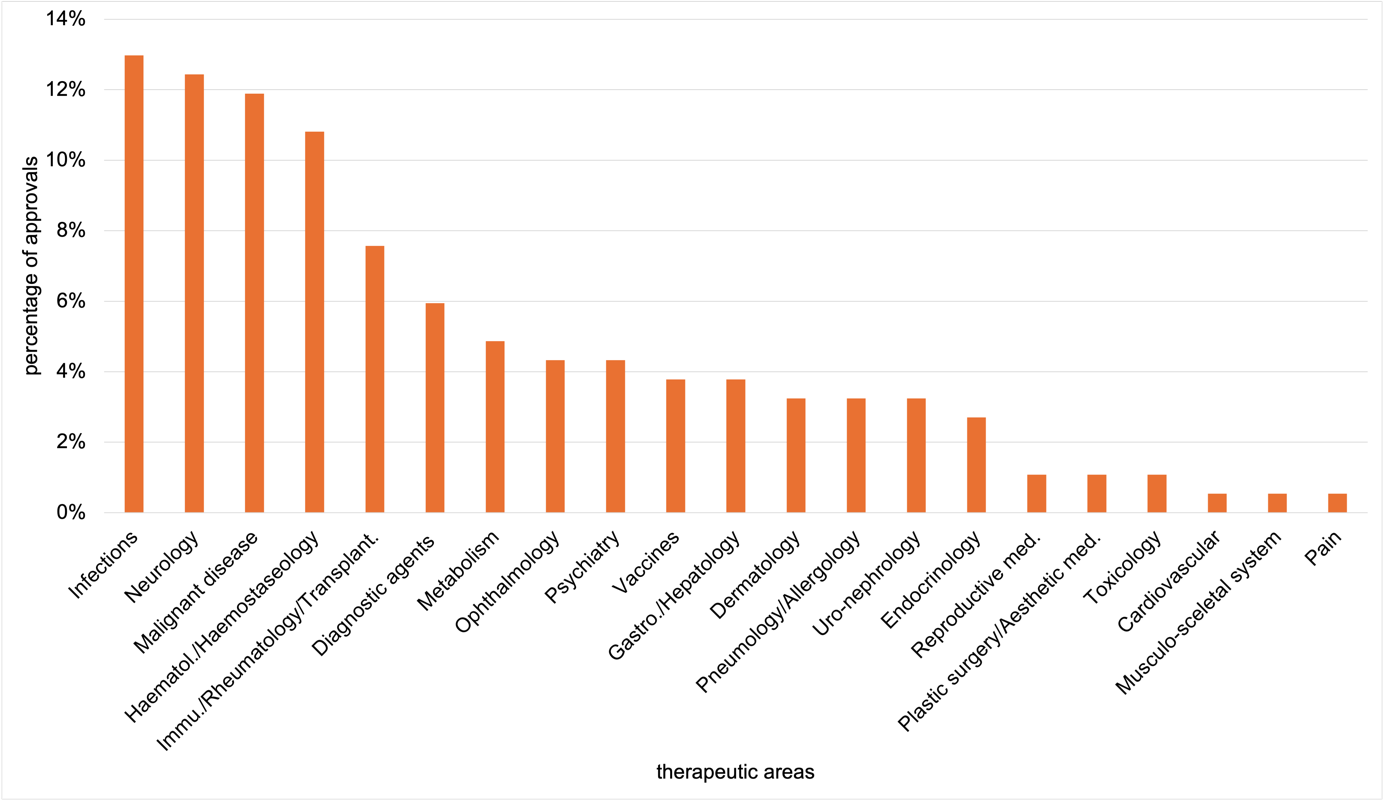
Fig. S3** Percentage distribution of FDA novel drug approvals by therapeutic areas from 2013 to 2023, shown in a bar chart


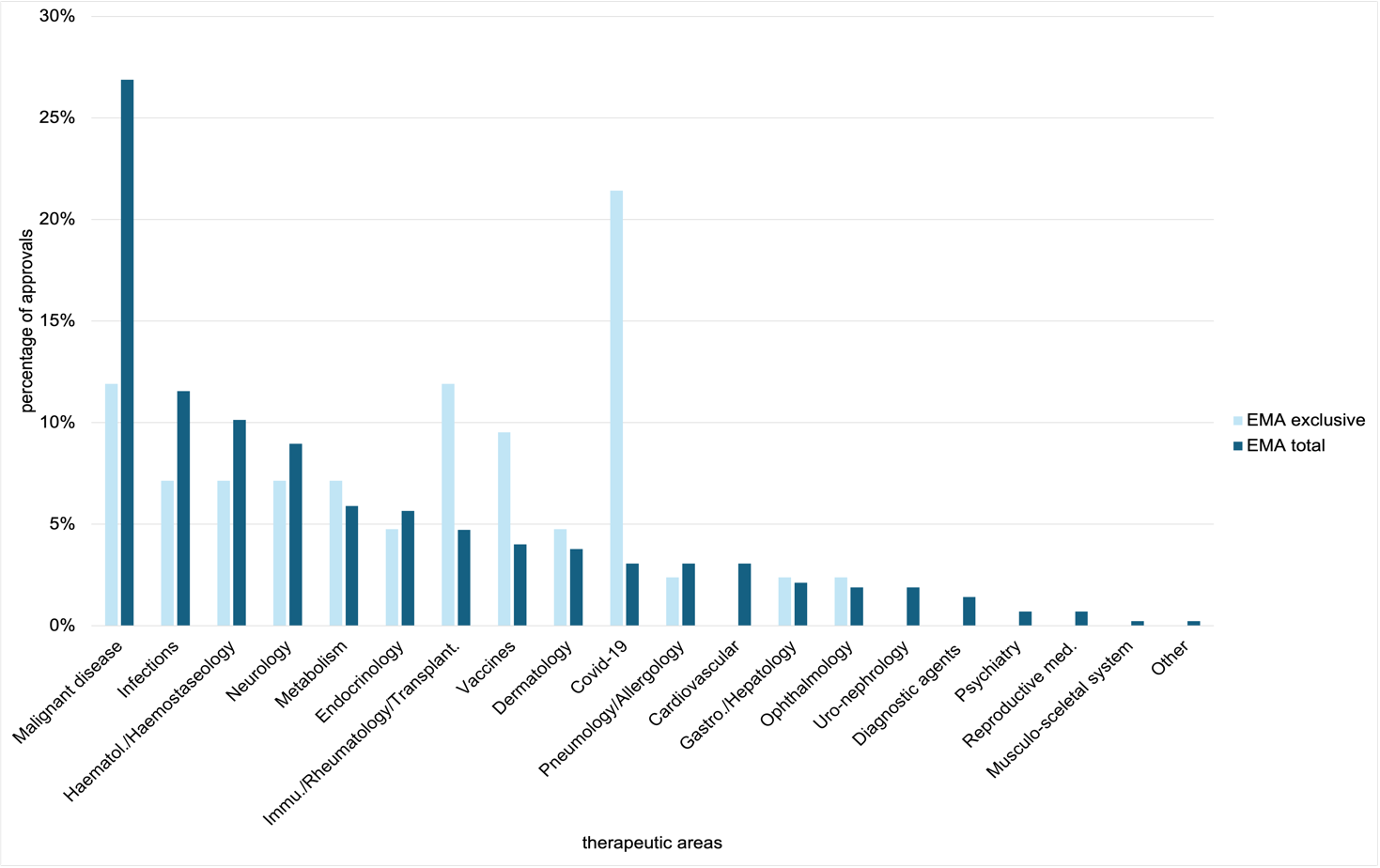
**Fig. S4** Percentage distribution of total and exclusive EMA approvals by therapeutic area from 2013 to 2023, shown in a bar chart


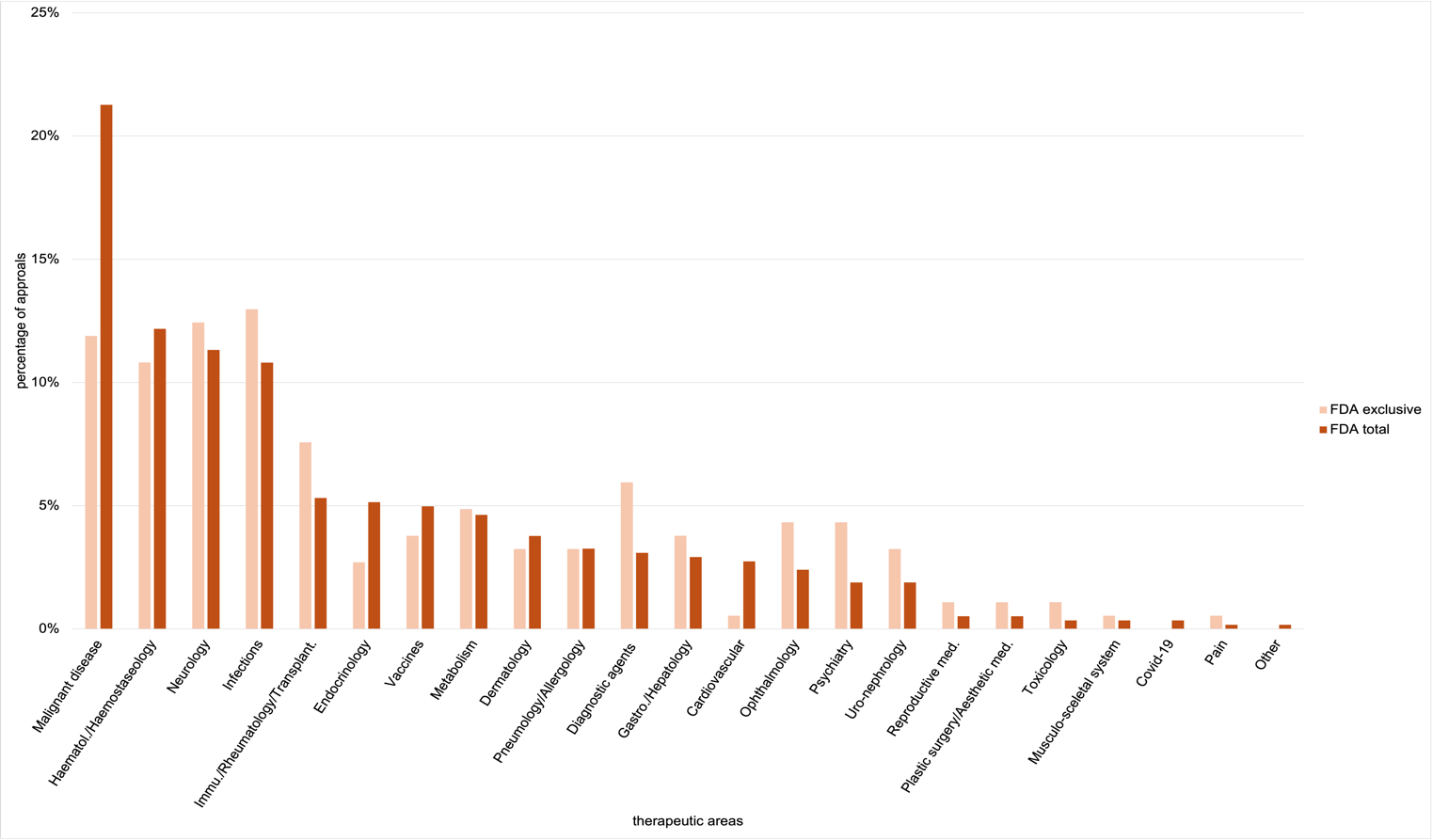


**Fig S5** Percentage distribution of total and exclusive FDA approvals by therapeutic area from 2013 to 2023, shown in a bar chart

**
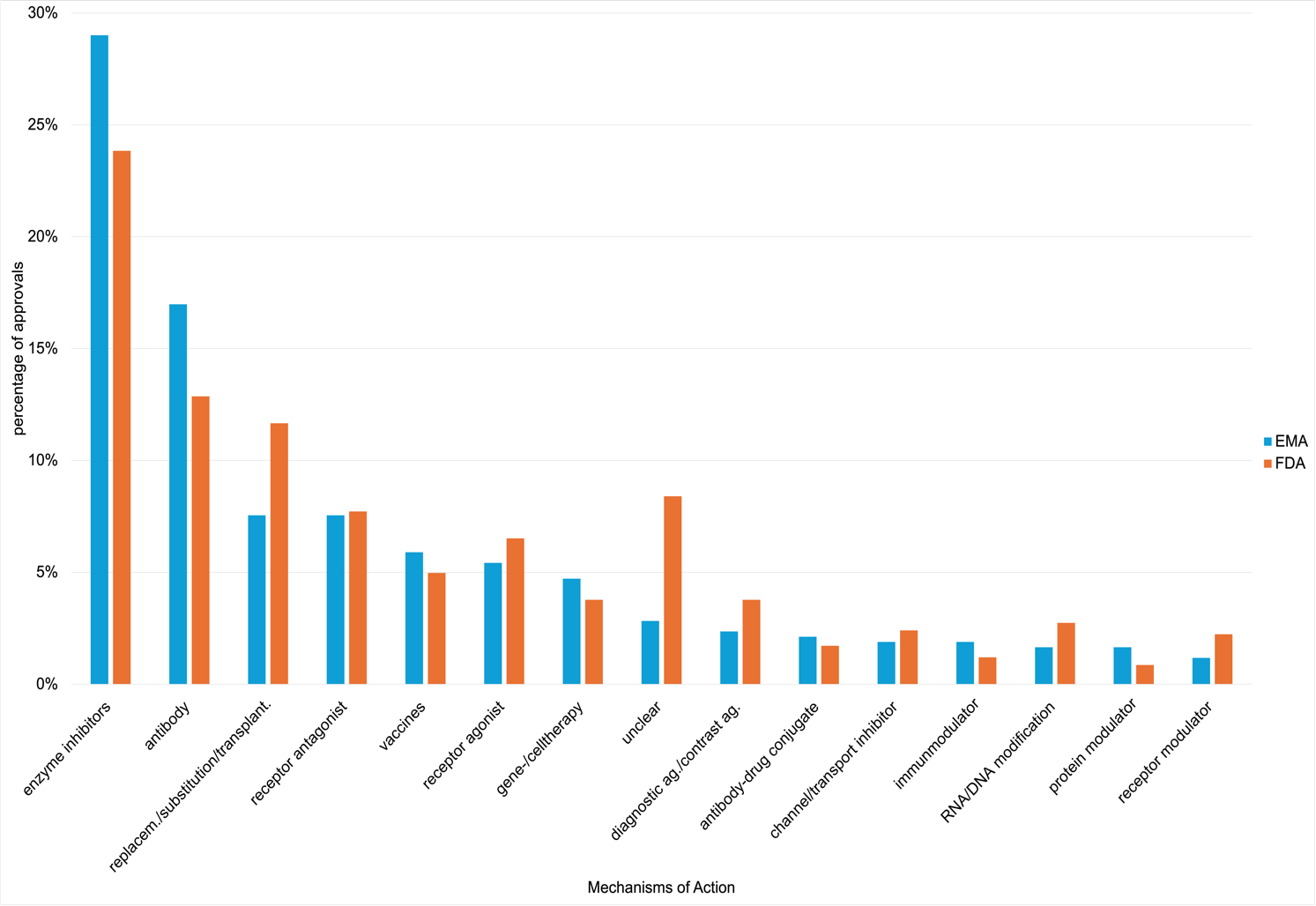
Fig. S6** Percentage distribution of EMA and FDA novel drug approvals by the top 15 mechanisms of action from 2013 to 2023, shown in a bar chart


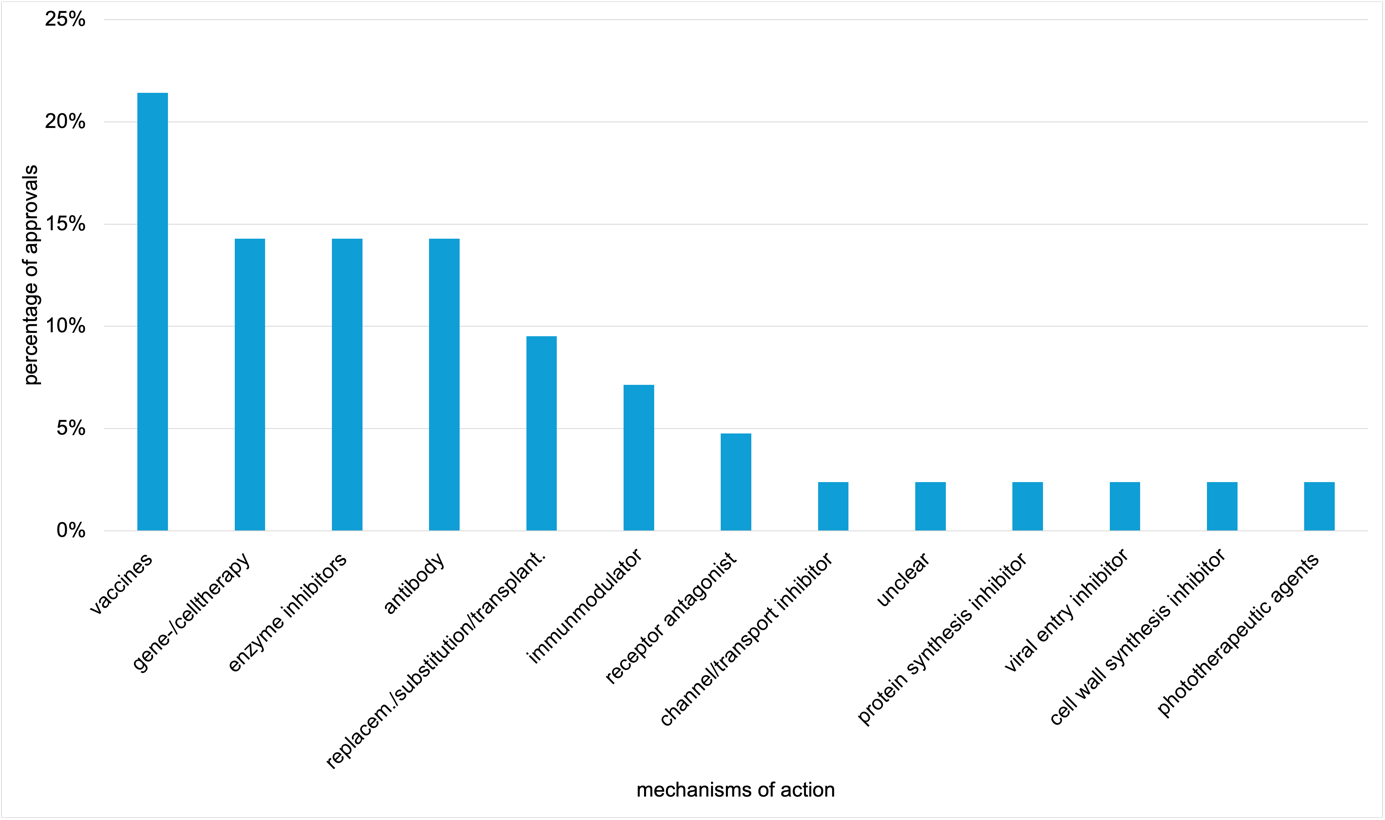
**Fig. S7** Percentage distribution of EMA novel drug approvals by mechanisms of action from 2013 to 2023, shown in a bar chart


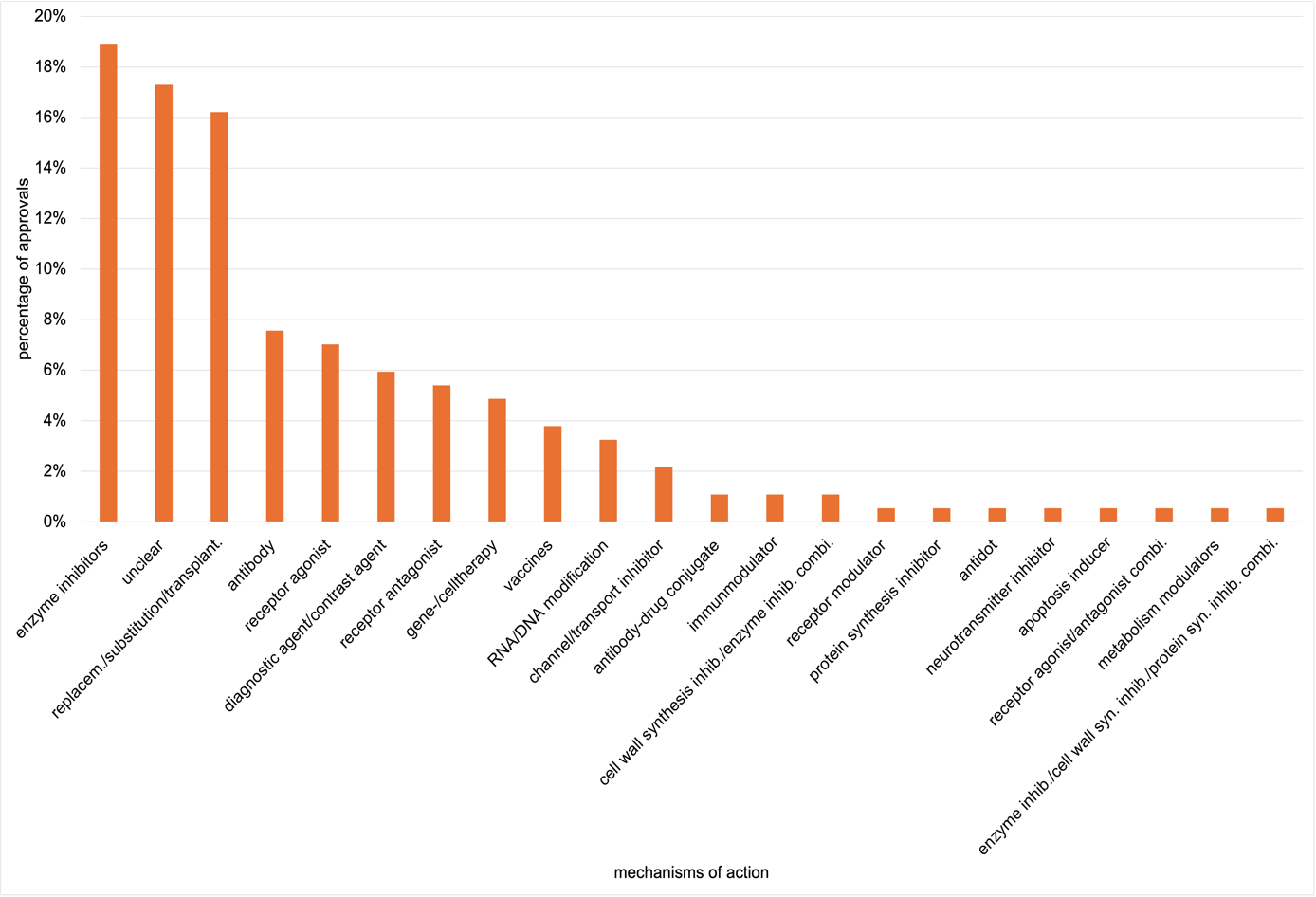
**Fig. S8** Percentage distribution of FDA novel drug approvals by mechanisms of action from 2013 to 2023, shown in a bar chart


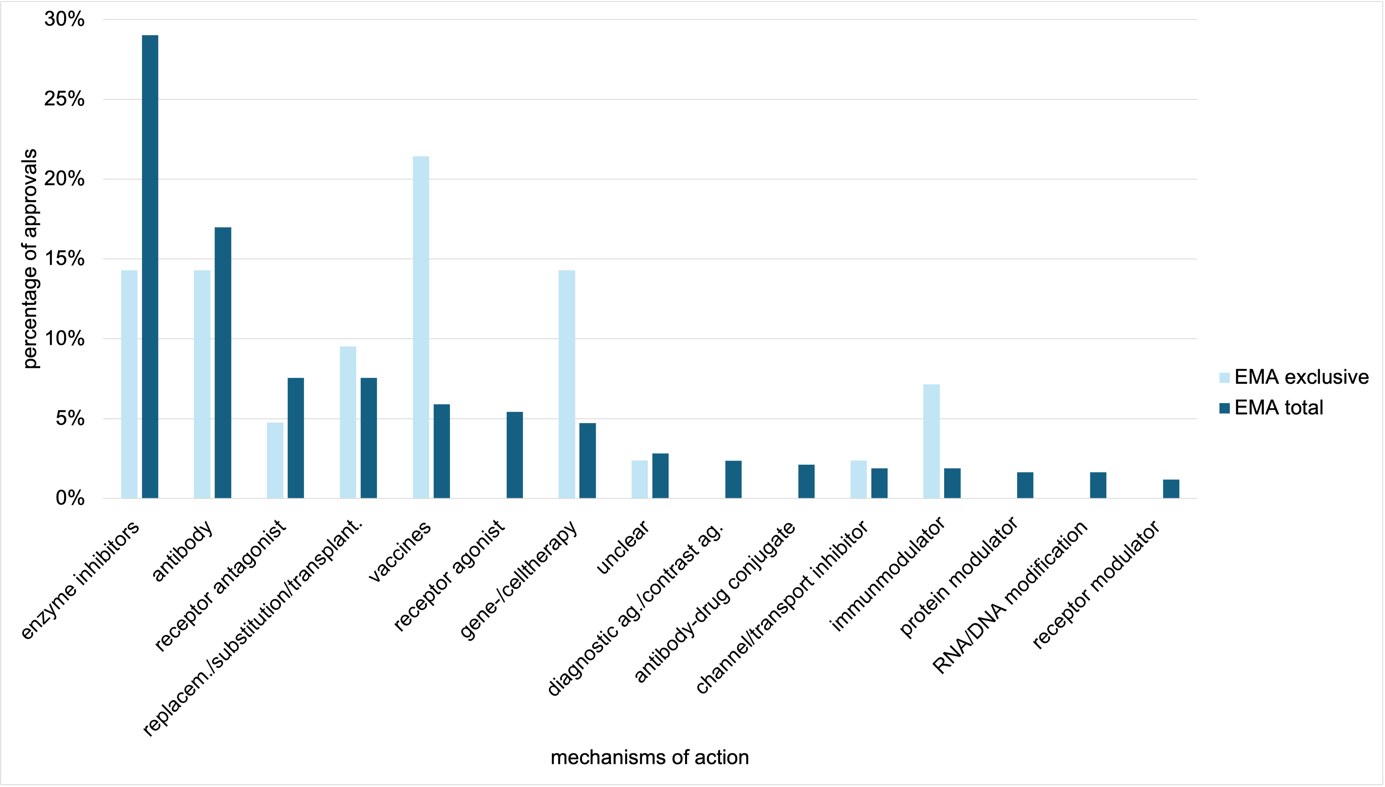
**Fig. S9** Percentage distribution of total and exclusive EMA approvals by top 15 mechanisms of action from 2013 to 2023, shown in a bar chart


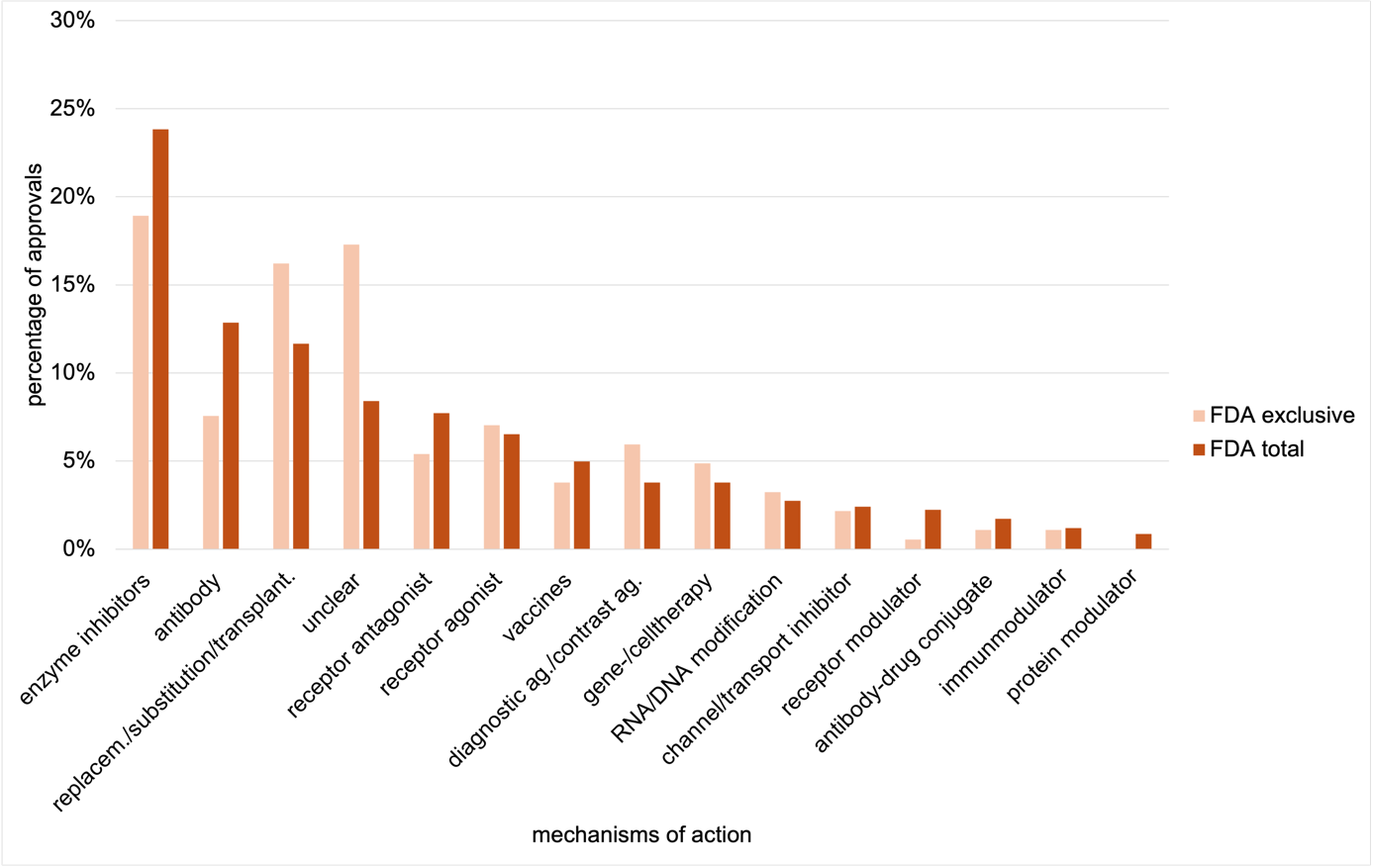
**Fig. S10** Percentage distribution of total and exclusive FDA approvals by top 15 mechanisms of action from 2013 to 2023, shown in a bar chart


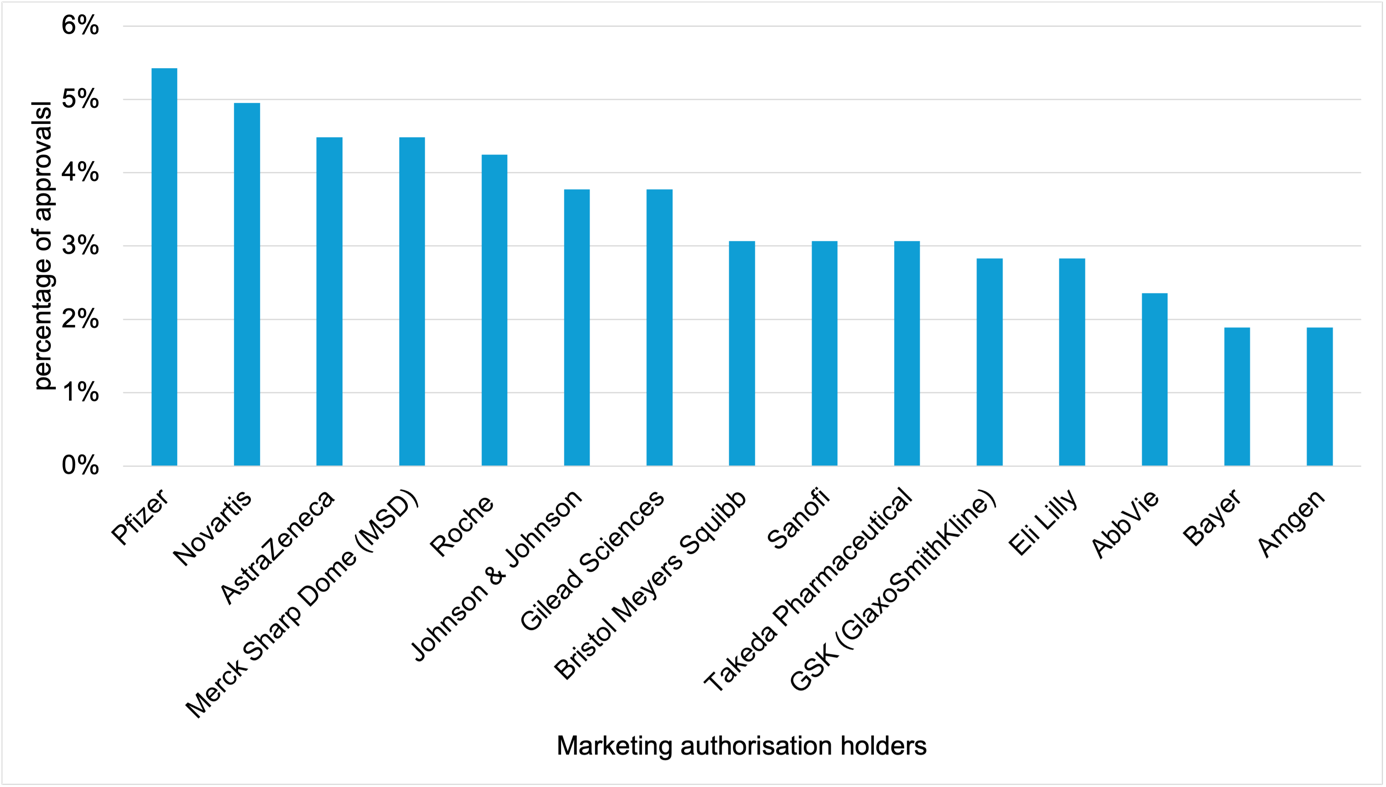


**Fig. S11** Percentage distribution of EMA novel drug approvals by the top 15 marketing authorisation holders from 2013 to 2023, shown in a bar chart


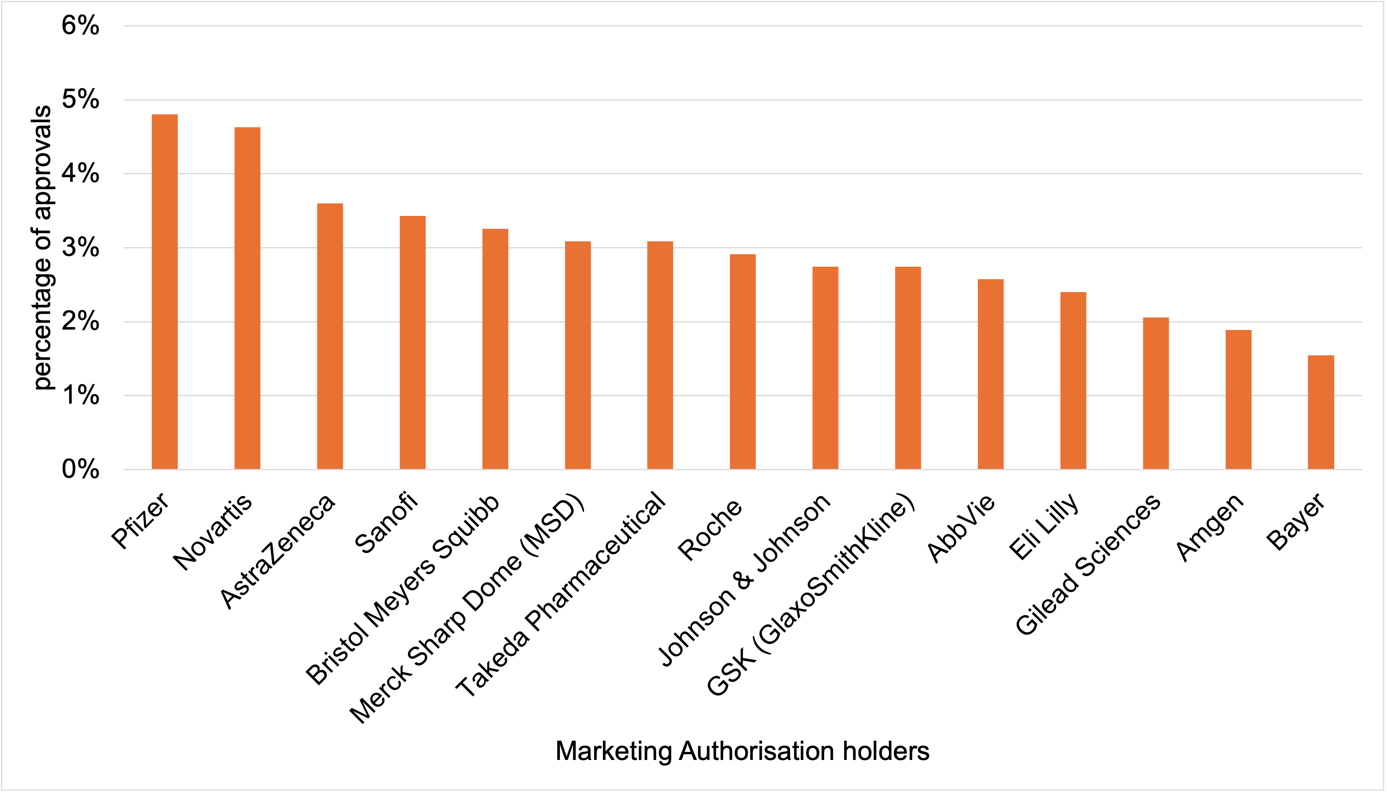


**Fig. S12** Percentage distribution of FDA novel drug approvals by the top 15 marketing authorisation holders from 2013 to 2023, shown in a bar chart


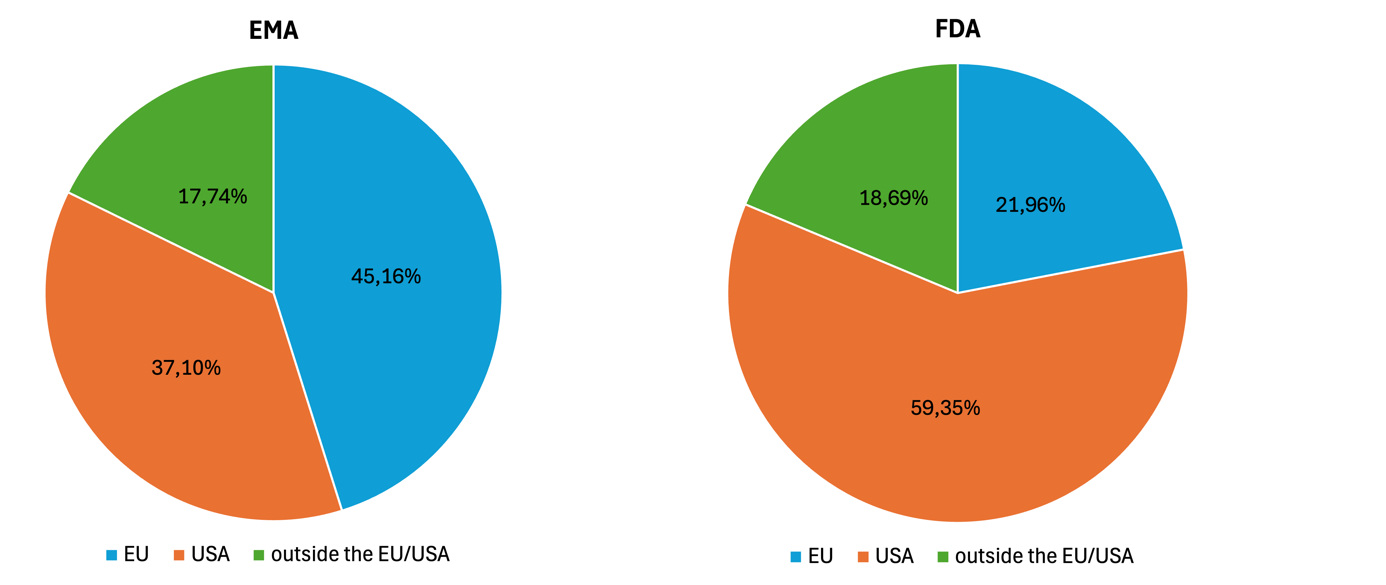


**Fig. S13** Percentage distribution of company headquarters of marketing authorization holders from 2013 to 2023, based on the total number of authorization holders, shown in two pie charts

**
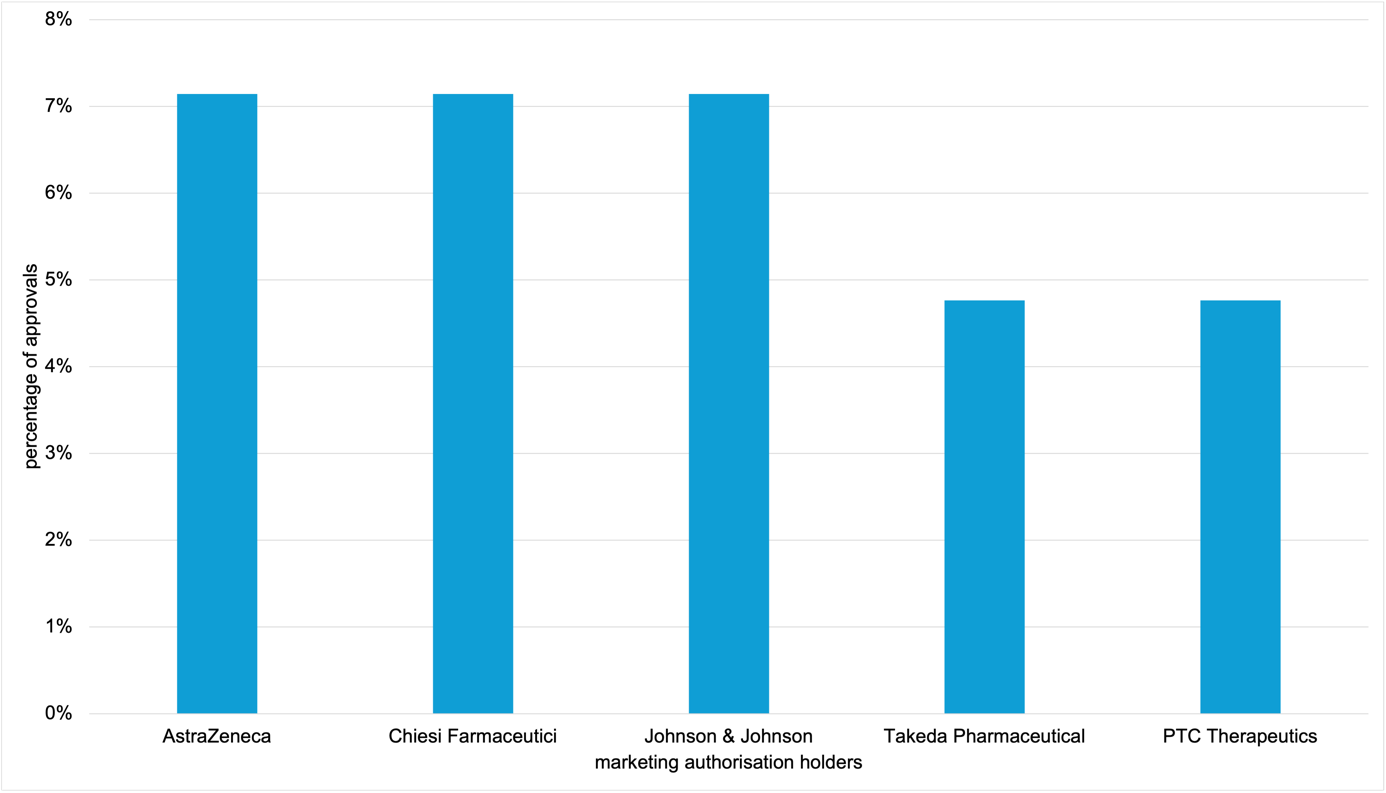
Fig. S14** Percentage distribution of EMA novel drug approvals by the top 5 marketing authorisation holders from 2013 to 2023, shown in a bar chart

**
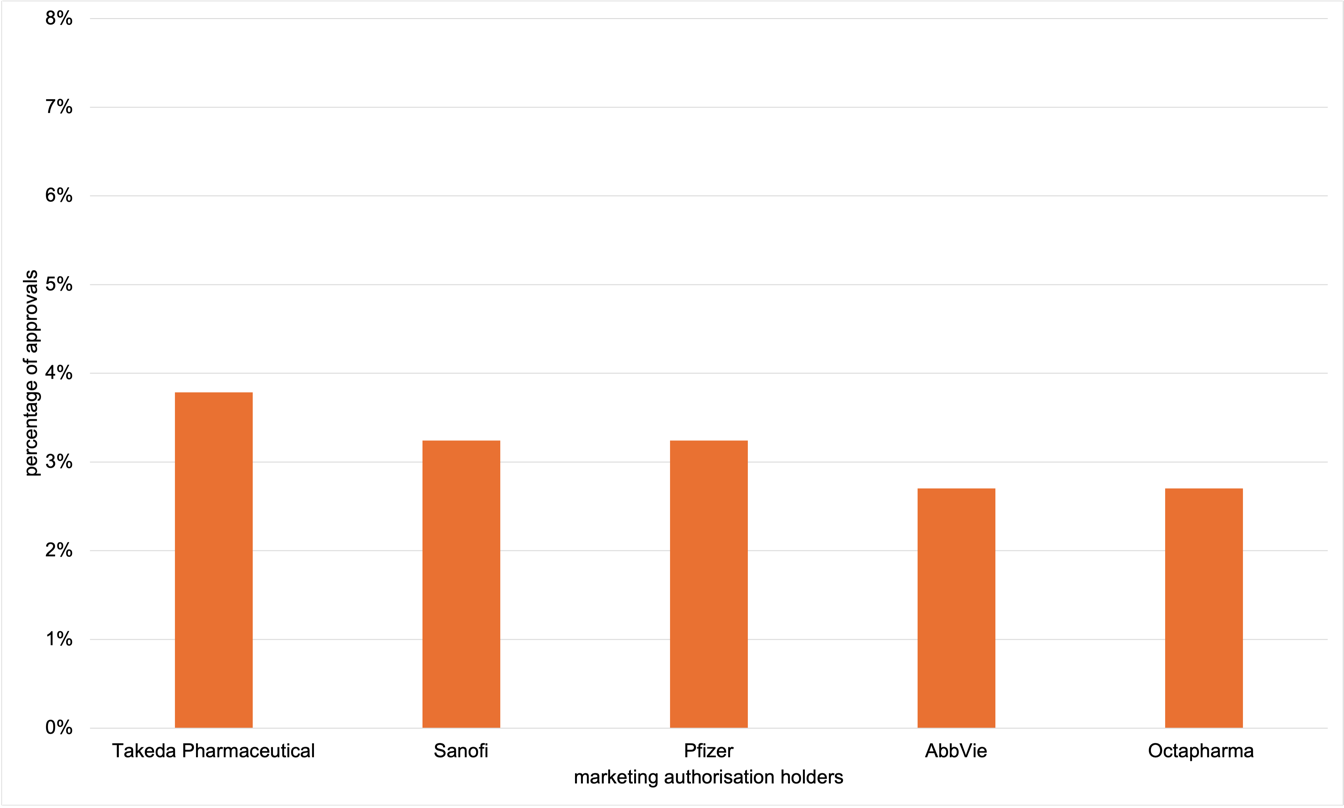
Fig. S15** Percentage distribution of FDA novel drug approvals by the top 5 marketing authorisation holders from 2013 to 2023, shown in a bar chart

**
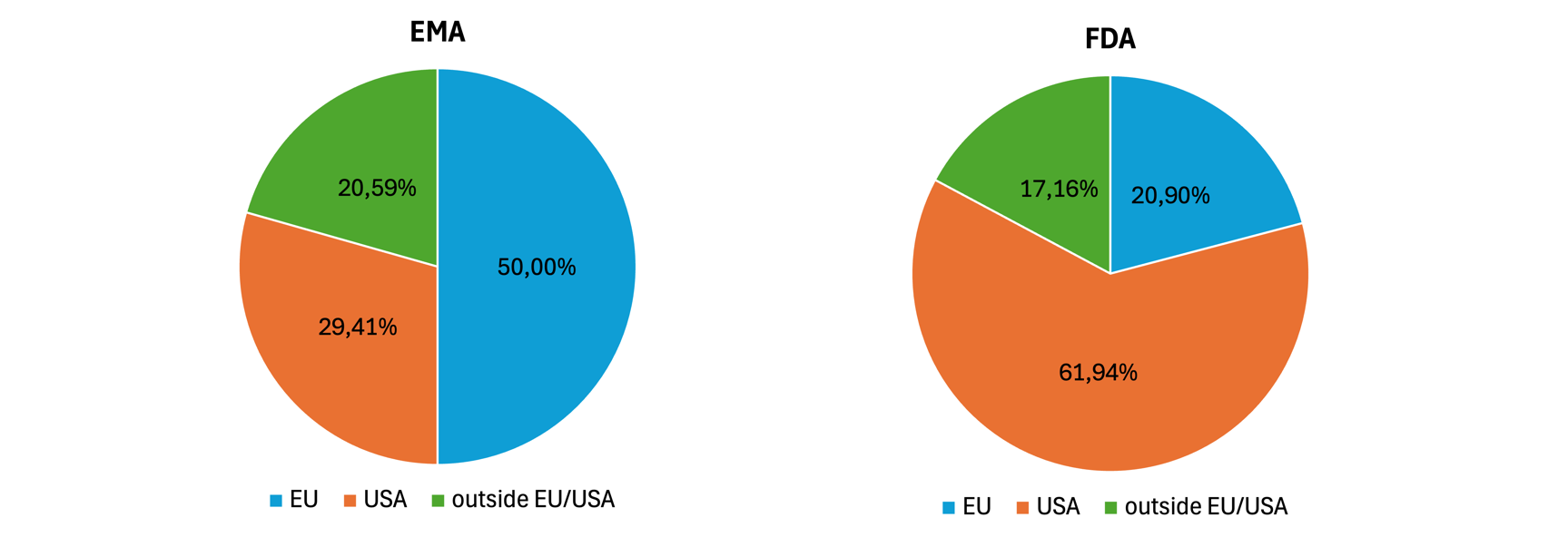
**

**Fig. S16** Percentage distribution of company headquarters of marketing authorization holders for exclusive drug approvals from 2013 to 2023, based on the number of authorization holders, shown in two pie charts
